# Circadian clock disruption promotes retinal photoreceptor degeneration

**DOI:** 10.1096/fj.202401967R

**Published:** 2025-04-02

**Authors:** Shumet T. Gegnaw, Cristina Sandu, Amandine Bery, Jacoline B. ten Brink, Nemanja Milićević, Aldo Jongejan, Perry D. Moerland, Arthur A. Bergen, Marie‐Paule Felder‐Schmittbuhl

**Affiliations:** ^1^ Centre National de la Recherche Scientifique Université de Strasbourg, Institut Des Neurosciences Cellulaires et Intégratives Strasbourg France; ^2^ Department of Human Genetics Amsterdam University Medical Centers, Location AMC, University of Amsterdam Amsterdam The Netherlands; ^3^ Faculty of Medicine and Health Technology Tampere University Tampere Finland; ^4^ Amsterdam UMC University of Amsterdam, Epidemiology and Data Science Amsterdam The Netherlands; ^5^ Amsterdam Public Health Methodology Amsterdam Amsterdam The Netherlands; ^6^ Amsterdam Institute for Immunology and Infectious Diseases Amsterdam The Netherlands; ^7^ Department of Ophthalmology Amsterdam University Medical Centers, Location AMC, University of Amsterdam Amsterdam The Netherlands; ^8^ Emma Centre for Personalized Medicine Amsterdam University Medical Centers, Location AMC, University of Amsterdam Amsterdam The Netherlands; ^9^ Present address: Department of Biotechnology, College of Natural and Computational Sciences, Debre Markos University Debre Markos Ethiopia

**Keywords:** *Bmal1*, circadian clock, ERG, P23H, retinitis pigmentosa, RNA‐seq, rod

## Abstract

Daily rhythms are a central hallmark of vision, in particular by adapting retinal physiology and light response to the day‐night cycle. These cyclic processes are regulated by retinal circadian clocks, molecular machineries regulating gene expression across the 24‐h cycle. Although hundreds of genes associated with genetic retinal disorders have been identified, no direct link has been established with the clock. Hence, we investigated the hypothesis that a poorly functioning circadian clock aggravates retinal photoreceptor disease. We performed this study in the P23H rhodopsin‐mutated mouse model (P23H Rho) that mimics one major cause of human autosomal dominant retinitis pigmentosa. We also used the rod‐specific knockout (rod‐*Bmal1KO*) of *Bmal1*, a key clock component. More specifically, we used either heterozygous P23H Rho mice or rod‐*Bmal1KO* alone, as well as double mutants of these strains and control mice. We showed by structural (histology, immunohistochemistry) and functional (electroretinography: ERG) analyses that the retinitis pigmentosa phenotype is exacerbated in the double mutant line compared to the P23H Rho mutation alone. Indeed, we observed marked ERG amplitude reduction and more photoreceptor cell loss in double mutants with respect to simple P23H Rho mutants. These observations were further corroborated by transcriptome analysis revealing major gene expression differences between these genotypes. In this data, we identified unique gene expression sets implicating neurogenesis, phototransduction cascade, and metabolism, associated with enhanced photoreceptor degeneration. Thus, our results establish a link between clock dysfunction and retinal degeneration and suggest underlying molecular mechanisms, together providing new concepts for understanding and managing blinding diseases.

## Graphical abstract

Mice carrying a knock‐in of the P23H mutation of the rhodopsin gene are a well‐characterized model of retinitis pigmentosa (RP), a hereditary retinal degeneration affecting primarily rods. Here, by combining the P23H Rho mutation with the rod‐specific invalidation of the *Bmal1* clock gene and by performing functional, structural, and molecular analyses, we show that clock impairment cell‐autonomously aggravates RP. Thus, a broken clock can constitute a compounding factor for retinal degeneration.

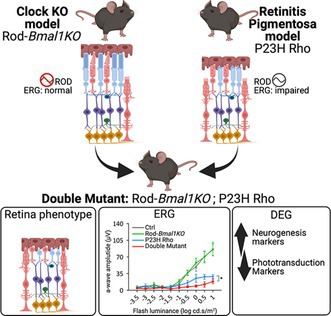

## 1 INTRODUCTION

Daily rhythms are a major hallmark of every cellular and physiological process in the body. These rhythms are programmed by molecular clocks widely distributed in mammalian cells and tissues. They are coordinated by a master clock located in the suprachiasmatic nuclei (SCN) within the hypothalamus. Circadian clocks keep time by using complex transcriptional/translational feedback loops involving transcription factors encoded by “clock genes” (mainly six core genes: *Bmal1*, *Clock*, *Per1*, *Per2*, *Cry1*, *Cry2*). They transmit their cycling patterns to their target genes, hence to cell physiology.^1^, ^2^

Genome‐wide transcriptome profiling studies in mouse have uncovered a wide array (about 43%) of protein‐encoding genes under circadian control. This seems to be in agreement with the need for different organs to fulfill distinct temporally controlled tasks.^3^ This study and similar others showed that the circadian clock system is highly pervasive, with extensive regulation of basic biological processes such as metabolism and DNA repair but also modulates tissue‐specific functions, all intimately coordinated within the 24‐h period.^4^, ^5^ Thus, circadian rhythmicity has strong adaptive value. It is, moreover, established that chronic disturbance of these timed mechanisms, as experienced in today's 24‐h lifestyles, leads to increased morbidity and reduced lifespan.^6^

In mammals, the retinal circadian clock plays a crucial function in adapting retinal physiology to the light/dark cycle (reviewed in Refs. [7, 8]). The retinal clock is currently considered a network of layer/cell‐specific oscillators^9^, ^10^ with expression of clock genes detected in most cell types.^11^, ^12^, ^13^ The circadian clock plays major roles in photoreceptor function and survival. These roles include the regulation, notably, of development,^14^, ^15^ of outer segment phagocytosis by the underlying pigmented epithelium^16^, ^17^, ^18^, ^19^ and response to phototoxicity.^20^, ^21^ In addition, light response and visual sensitivity display daily variations both under dark‐adapted^22^, ^23^ and light‐adapted conditions,^24^ that were shown to be disrupted in circadian clock gene mutants.^11^, ^25^, ^26^ Mutations in clock genes were also reported to impair retinal health. For instance, the mutation of *Bmal1* is known to reduce photoreceptor viability upon aging^27^, ^28^ but no direct link has been established so far with retinal diseases.

Retinitis pigmentosa (RP) is a rare inherited form of progressive retinal degeneration, affecting 1 in 4000 people.^29^, ^30^ The symptoms common to all RP types involve night blindness and progressive concentric visual field loss leading to tunnel vision. This is due to the initial death of rods, which subsequently triggers the degeneration of cones, frequently resulting in legal blindness.^30^ RP is a clinically and genetically heterogeneous disorder. Since the identification of the first mutation (P23H in rhodopsin gene) responsible for autosomal dominant RP,^31^ mutations in more than 90 genes have been implicated.^32^ Interestingly, individuals with the similar RP disease gene mutation can have different retinal phenotypes, likely due to unidentified genetic and/or environmental factors.^30^ Among the environmental candidate factors, a potential role of temporarily or chronically disturbed circadian rhythms has not been investigated up to now.

The present study investigated the hypothesis that a poorly functioning circadian clock affects retinal function and negatively modulates the disease phenotype seen in RP. We tested our hypothesis by crossing *Rho* ^*P23H/+*^ mouse,^33^, ^34^ a well‐established model of human RP, with the rod‐specific *Bmal1* knockout (KO), that displays no rhythm in dark‐adapted light response.^11^ We analyzed the double mutants and controls by electroretinogram (ERG), histology, immunohistochemistry, and whole retina RNA‐sequencing. Experimental results support our hypothesis: we found that the conditional, rod‐specific, KO of *Bmal1* markedly exacerbates retinal RP phenotypes induced by the P23H mutation of rhodopsin and induces accelerated death of rods and cones.

## 2 MATERIALS AND METHODS

### 2.1 Animals

All experimental procedures were carried out according to the Directive of the European Parliament and The Council of the European Union (2010/63/EU) for animal experiments and authorized by the French Ministry for Higher Education, Research and Innovation (APAFIS#10213‐2 017 060 920 001 367‐v3). *Rho* ^*P23H/P23H*^, *Rho‐iCre*, and *Bmal1* ^*fl/fl*^ mice were purchased from Jackson laboratories (Bar Harbor, ME, USA). All mice (males and females) had a C57BL/6J background, the absence of the *rd8* mutation being previously validated.^35^ Animals were bred in the Chronobiotron animal facility (UAR 3415, CNRS‐University of Strasbourg) on a 12 h–12 h light–dark (LD) cycle (ZT0—light on, ZT12—light off; 300 lx during the light phase, dim red light <5 lx during the dark phase) in an ambient temperature of 22 ± 1°C, with free access to food and water.

The study was performed on distinct genotypes generated by breeding mice carrying the P23H mutation of rhodopsin (*Rho* ^*P23H/P23H*^),^34^ the floxed *Bmal1* allele (*Bmal1* ^*fl/fl*^),^26^ and Rhodopsin‐iCre (*Rho‐iCre*) transgene.^36^ We generated four main genotype groups. For homogeneity and in order to use a maximum of littermates, all groups contained the *Rho‐iCre* transgene and at least one floxed allele of *Bmal1*. The four groups were: (1) the double mutant carrying both the heterozygous P23H mutation and the rod‐specific deletion of *Bmal1* (DM: *Rho* ^*P23H/+*^ *; Bmal1* ^*fl/fl*^ *; Rho‐iCre/+*); (2) a simple mutant with only the heterozygous P23H mutation (P23H Rho: *Rho* ^*P23H/+*^ *; Bmal1* ^*fl/+*^ *; Rho‐iCre/+*); (3) a simple mutant with only the rod‐specific deletion of *Bmal1* (rod‐*Bmal1KO*: *Rho* ^*+/+*^ *; Bmal1* ^*fl/fl*^ *; Rho‐iCre/+*); (4) control mice with no mutation in rhodopsin but with *Rho*‐*iCre* transgene and one floxed *Bmal1* allele (Ctrl: *Rho* ^*+/+*^ *; Bmal1* ^*fl/+*^ *; Rho‐iCre/+*). In Ctrl mice, the circadian clock in rods is unaltered.^11^, ^37^ We initially confirmed that the *Rho‐iCre* transgene and/or knockout of one *Bmal1* allele did not induce any specific phenotype by comparing visual responses (ERG) of the Ctrl group to mice carrying uniquely one floxed allele of *Bmal1* or carrying uniquely the *Rho‐iCre/+* transgene (Figure S1 and Table S1). All mice were also bred on the *mPer2* ^*Luc*^ clock reporter background^38^ to evaluate whether the distinct genotypes impact the retinal clock (Figure S3).

Mice were genotyped by PCR on tail genomic DNA. Genotyping of the *Bmal1* conditional knockout mice was performed as previously described.^26^ The primers^11^ to detect the *Rho‐iCre* transgene were: 5′‐AGCAGCCTTGGTCTCTGTCTAC‐3′ and 5′‐GATTCTCCTCATCACCAGGGAC‐3′. For the detection of the P23H knock in, we used: 5′‐GCCTGTTTAGCTGAGAAAAC and 5′‐GACCACGTAACAAACTTCTG‐3′. Genotyping for the *Per2‐luc* allele was performed as described.^38^

### 2.2 Electroretinography (ERG)

Scotopic ERG recording was performed according to previously described procedures on animals that were dark‐adapted since lights off in the preceding evening.^39^, ^40^ Animal handling before recording was performed under dim red light. ERG recordings were made in the afternoon (CT7 to CT10: CT0 stands for the time of lights on during the preceding day) and genotypes were randomly allocated to avoid bias from circadian changes in visual responses. We chose to perform these recordings during subjective daytime to avoid any confounding effect from circadian time, since the response of rod‐*Bmal1KO* mice in the daytime is similar to that of the Ctrl.^11^ Prior to ERG recording, we made sure that the distinct animal groups were properly synchronized to the LD cycle (Figure S2) and that their retinas had intact rhythmicity, as assessed by recording rhythms in PER2::LUCIFERASE activity in vitro (Figure S3). This was in agreement with our previous findings,^11^, ^41^ confirming that circadian clock impairment specifically in rods does not markedly impact the oscillator network in the retina.

In summary, the ERG device consisted of a Ganzfeld bowl, an amplifier, and a PC‐based control and recording unit (RETI port/scan 21; Stasche & Finger GmbH, Roland Consult, Brandenburg, Germany). Mice were anesthetized with a combination of xylazine (15 mg/kg, Rompun 2%; Bayer, Puteau, France) and ketamine (75 mg/kg, Imalgene 1000; Merial, Lyon, France) and placed on a temperature‐regulated heating plate. Ground and reference electrodes were placed subcutaneously in the base of the tail and below ear lobes (right and left), respectively. ERG was recorded from both eyes using corneal/active electrodes (thin gold‐wire with a 2‐mm ring end). Ocrygel (Virbac, Carros, France) eye drop was applied to ensure good electrical contact and to keep the cornea hydrated during the entire procedure. The following scotopic white flash ascending intensities were used: 3 × 10^−4^, 10^−3^, 3 × 10^−3^, 10^−2^, 3 × 10^−2^, 10^−1^, 3 × 10^−1^, 1, 3, and 10 cd.s/m^2^. Animals were then light‐adapted for 10 min under background illumination before recording light responses to 10 cd.s/m^2^ flashes of white light.

Data were treated according to standard procedures.^40^ For the extraction of oscillatory potentials (OPs), recording data at 10 cd.s/m^2^ in scotopic conditions were bandpass‐filtered between 55 and 280 Hz, and the amplitudes of the four OPs summed.^25^

Statistical analysis of ERG data was performed using Sigma Plot 13 and GraphPad Prism 10. Due to the non‐normality of the data (Shapiro–Wilk's test), Mixed‐effects model analysis was performed for ERG a‐ and b‐wave amplitudes within age groups, with light intensity and genotype as factors. Light intensity and genotype exhibited a significant effect on a‐ and b‐wave amplitudes, throughout all age groups. Comparison of P23H Rho and DM genotype groups was also performed by Mixed‐effects model analysis. Amplitudes of oscillatory potentials were compared within age groups by one way analysis of variance or Kruskall‐Wallis Anova on ranks and, respectively, Holm‐Sidak or Dunn's post hoc testing. Results are presented as means ± SEM and the statistical significance level was set at *p* < .05.

### 2.3 Retinal histology and immunohistochemistry

Mice were euthanized by cervical dislocation. Retinas from left eyes were quickly dissected away from the eyecup in ice‐cold HBSS buffer within a petri dish, snap‐frozen on dry ice, and stored at −80°C. Right eyes were quickly removed and immersion‐fixed in 4% paraformaldehyde in phosphate‐buffered saline (PBS) overnight at 4°C, then transferred into fresh PBS (with 0.05% sodium azide) and stored at 4°C until needed for histological and immunohistochemical analysis.

For histological analysis, dissected eyecups were dehydrated in an ascending ethanol series, transferred to toluene, and embedded in paraffin wax. Transversal sections (5 μm) were prepared on a microtome, and those close to the optic nerve were collected on microscope slides. Deparaffinized sections were stained with Carazzi's hematoxylin solution for 5 min, followed by counterstaining with Eosin Y for 30 s (H&E staining). The stained slides were scanned in a NanoZoomer S60 Digital slide scanner C13210 (Hamamatsu) at 40× in bright field. Images were in NanoZoomer Digital Pathology Image (ndpi) format and viewed in NDP.view 2 for morphological analysis. Photoreceptor nuclei were counted all along the retina, from the optic nerve head (ONH) to the periphery. Rectangles covering a 100 μm width were placed over the image of retinal sections from the ONH to the periphery and nuclei were counted in the ONL within the boundaries of the rectangle by using QuPath^42^ (*n* = 3–4 per genotype). Data were analyzed by Two‐way repeated measures ANOVA and Holm‐Sidak's post hoc test. The thickness of the inner + outer segments, of the inner nuclear layer (INL) and of the outer and inner plexiform layers was measured at 750 μm of the ONH. Nuclear density of the inner and ganglion cell layers was also measured at 750 μm of the ONH within a 100 μm‐width rectangle. Data were analyzed by One‐way ANOVA and Holm‐Sidak's post hoc test.

Immunohistochemistry analysis of retinas was performed as previously described.^43^ In summary, cryostat sections (10 μm) were permeabilized with Triton (PBS‐0.1% Triton X‐100, 5 min) and blocked with saturation buffer (3% bovine serum albumin, 0.05% Tween‐20, and 0.1% sodium azide in PBS) for 60 min. Sections were incubated overnight with primary antibodies diluted in saturation buffer, washed 2 × 5 min in PBS, incubated with secondary antibodies (2 h at room temperature) and washed again for 40 min. Cell nuclei were stained with 4,6‐di‐amino‐phenylindol amine (DAPI; Molecular Probes). Slides were washed 3 × 5 min in PBS, mounted in PBS/glycerol (1:1), and scanned in a NanoZoomer (Hamamatsu Photonics) at 40× and viewed using NDP.view2 software (Hamamatsu Photonics). Immunostaining was performed on four consecutive sections per eye (*n* = 4 animals per genotype). Primary antibodies used for immunolabeling were monoclonal anti‐rhodopsin rho‐4D2,^44^ polyclonal anti‐cone arrestin (Ref. [45] a kind gift from Cheryl Craft, dilution 1:500), polyclonal anti‐PKCα (ab32376, Abcam, dilution 1:500), and polyclonal anti‐glial fibrillary acidic protein (GFAP) (z0334, Dako, dilution 1:500). Secondary antibodies were Alexa goat anti‐mouse or anti‐rabbit 488 and goat anti‐rabbit 594 (Thermofisher, dilution 1:500).

### 2.4 RNA extraction, RNA‐seq library preparation and RNA sequencing

RNA sequencing was performed on retinas from five genotype groups (*n* = 4 per genotype); Ctrl, rod‐*Bmal1KO*, P23H Rho, DM, and an additional group of mice containing one floxed allele of *Bmal1* (*Bmal1* ^*fl/+*^). Sampled retinas were individually homogenized using a motorized pestle in 500 μL TRI Reagent (Molecular Research Center, Cincinnati, OH, USA) and incubated for 5 min at room temperature. Chloroform (100 μL) was added to each lysate, and after 2 min incubation at room temperature, the mixture was centrifuged using Phase‐Lock gel tubes (Heavy, 2 mL; QuantaBio, Beverly, MA, USA) at 12 000 g for 15 min at 4°C. The RNA from the upper aqueous phase was precipitated with an equal volume of 70% ethanol and extracted using the RNeasy Micro kit (Qiagen GmbH, Hilden, Germany) following the manufacturer's instructions, including the DNase digestion step to remove any genomic DNA contamination. The RNA was eluted with 14 μL of RNase‐free water. RNA concentration and purity were measured using a NanoDrop ND‐1000 V 3.5 Spectrophotometer (NanoDrop Technologies, Wilmington, DE, USA), and its quality was evaluated with a Bioanalyzer (AgilentTechnologies, Amstelveen, The Netherlands) (RNA integrity numbers were between 7.4‐10).

RNA‐seq library preparation was performed exactly as described previously.^14^ Briefly, libraries were prepared from 500 ng purified RNA using the KAPA mRNA HyperPrep Library Preparation Kit (Roche Sequencing Solutions, Pleasanton, CA USA) for Illumina Platform HiSeq 4000, according to the manufacturer's protocol. The libraries were amplified using a mixture of KAPA HiFi HotStart RdyMix (2×) and Lib. Amp. Primer Mix (10×) (Roche Sequencing Solutions, Pleasanton, CA, USA) to produce strand‐specific PCR products. The cDNA library amplification process was confirmed using flash gel visualization along with cleanup steps. Quality and size distribution of the cDNA library were checked by using the Bioanalyzer. Qubit 2.0 Fluorometer (Life Technologies, Foster City, CA, USA) was used for the quantification of libraries. The cDNA library was sequenced by single‐end (50 bp) sequencing on Illumina HiSeq 4000 sequencer (Illumina, San Diego, CA, USA).

### 2.5 Pre‐processing and analysis of RNA‐seq data

Reads were subjected to quality control using FastQC v0.11.5, and trimmed for adapter sequences using Trimmomatic v0.36. The trimmed reads were aligned against the reference mouse genome (Ensembl GRCm38.p7) using HISAT2 (v2.1.0).^46^ Counts were obtained using HTSeq (v0.6.1)^47^ with parameters “‐‐stranded=reverse ‐‐order=name ‐‐minaqual=10 ‐‐type=exon ‐‐idattr=gene_id ‐‐mode=union” and the mouse GTF from Ensembl GRCm38v93.

Statistical analyses were performed using the edgeR^48^ and limma^49^ R/Bioconductor packages using R (v3.6.0) and Bioconductor (v3.9). 23 905 genes with more than 2 count‐per‐million reads (CPM) in at least 4 samples were retained. Count data were transformed to log2‐counts per million (logCPM), normalized by applying the trimmed mean of M‐values method^48^ and precision weighted using voom.^50^

The differential expression between the 5 distinct genotypes was evaluated using a moderated t‐test within limma's linear model framework while correcting for gender. Resulting *p*‐values were corrected for multiple testing using the Benjamini‐Hochberg false discovery rate. Resulting adjusted (Adj.) *p*‐values ≤ .05 were considered statistically significant. Additional gene annotation was retrieved from Ensembl (v97) using the biomaRt R/Bioconductor package.

Gene ontology (GO) and pathway enrichment analyses were performed using g:Profiler^51^ with the *Mus musculus* transcriptome as the reference background. An adjusted *p*‐value < .05 was set as the threshold for significantly enriched pathways using the g:SCS method to correct for multiple testing. We investigated interactions between protein products from the list of genes up‐or down‐regulated in the DM with respect to P23H Rho by STRING analysis, with the threshold for the minimum required interaction score set at 0.3–0.7 [STRING version 12 (https://string‐db.org/)^52^].

### 2.6 Analysis of non‐coding regulatory sequences

Selection of conserved mouse/human non‐coding elements (CNEs) located in introns, 5′ and 3′ regions up to the neighbor gene as defined in UCSC (genomes mm10 and hg19, respectively) were retrieved using the “comparative genomics” and “intersection” tools in UCSC browser and Galaxy (Ref. [53]; https://main.g2.bx.psu.edu/). CNEs were kept for further analysis if they contained either a promoter or an enhancer‐like signature (related to H3K4me3 or H3K27ac) or a CTCF‐binding site according to ENCODE Candidate Cis‐Regulatory Elements (cCREs).^54^, ^55^, ^56^ The CNEs were analyzed when the conservation to 60 vertebrate species (Multiz60way) was at least 80%.

Searches for enriched motifs within selected lists of CNEs were performed using the web server TFmotifView,^57^ with “enrichment” threshold >1.5 (i.e., a given motif is present 1.5 times more in the foreground than in the background), and with a *p*‐value cut‐off at .05. The “foreground” corresponded to the selected CNEs and the “background” to shuffled foreground sequences, based on basic permutation methods with conservation of nucleotide counts but randomized order of arrangement.

### 2.7 RT‐qPCR analysis to validate results from RNA sequencing

Analysis was performed as described previously.^14^ 150 ng total RNA from whole retinas was submitted to cDNA synthesis (iScript Advanced cDNA synthesis Kit, Biorad, USA) and qPCR by the TaqMan technology (Thermo Fisher, France) with the following Gene Expression Assays for target genes (*Bmal1*: Mm00500226_m1, *Rho*: Mm01184405_m1, *Efna5*: Mm01237700_m1 and *Lhx3*: Mm01330619_g1) and housekeeping genes (*Sdha*: Mm01352363_m1 and *Hprt*: Mm01324427_m1). Statistical analysis of relative expression levels was performed by One‐way ANOVA and Holm‐Sidak's post hoc testing (Sigmaplot V12, Systat Software Inc.).

## 3 RESULTS

### 3.1 Rod‐specific deletion of *Bmal1* exacerbates visual dysfunction induced by the P23H rhodopsin mutation

**FIGURE 1:**
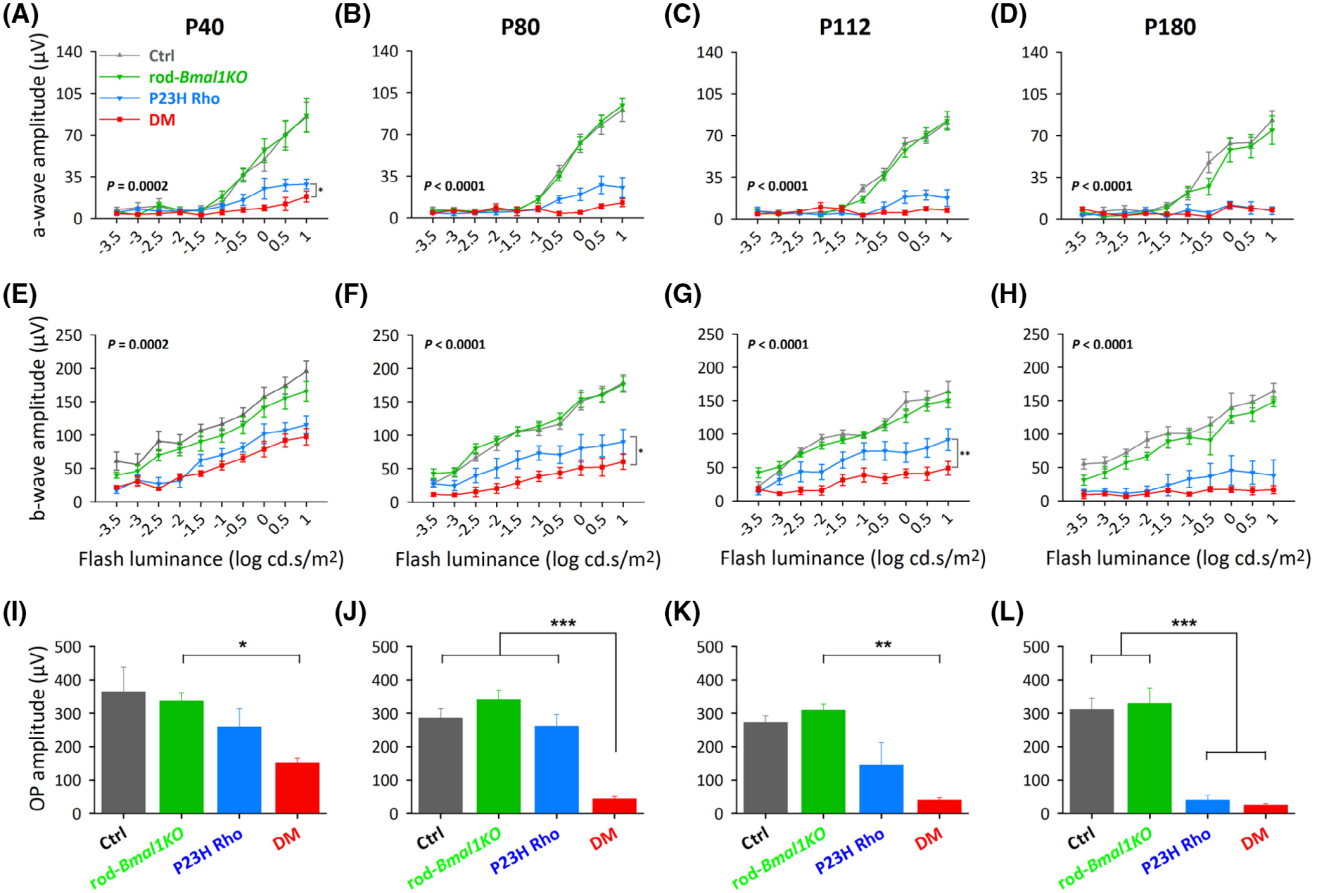
Dark‐adapted ERG analysis reveals synergistic effect between the simple P23H mutation of rhodopsin and the rod‐specific *Bmal1* KO. Amplitudes of a‐ and b‐waves are presented according to intensity of the light stimulus in Ctrl (gray), rod‐*Bmal1KO* (green), P23H Rho (blue), and DM (red) groups at P40 (A, E), P80 (B, F), P112 (C, G) and P180 (D, H). There is a genotype effect (*p*‐value on the graphs) at all ages, on a‐ and b‐wave amplitudes (Mixed‐effects model analysis). (A–D) a‐wave in DM displayed a tendency for reduced amplitude with respect to littermate P23H Rho mice at all ages and was significantly lower at P40 (*p* < .05, A). (E–H) b‐wave amplitudes in DM mice appear also reduced with respect to P23H Rho. This decrease is significant at P80 (*p < .05, F) and P112 (**p < .01, G). No more difference is detectable between DM and P23H Rho mice at P180 (D, H), with nearly undetectable visual responses in both genotypes. Comparison between genotypes was performed by Mixed‐effects model analysis and Holm‐Sidak's post hoc testing (*n* = 6–10, *n* = 6–8, *n* = 5–8, and *n* = 5–6/genotype at P40, 80, 112, and 180, respectively). (I–L) Analysis of oscillatory potentials (OP) amplitudes (sum of amplitudes from the four OPs) shows a significant decrease in the light‐response signal originating from the inner retina in the DM already at P40 (I), progressively declining until P180 (I–L). Comparison between genotypes was performed by One way analysis of variance (*n* = 4 to 8). Data are means ± SEM. *p < 0.05, **p < 0.01, ***p < 0.001.

We performed dark‐adapted ERG to evaluate visual function and potential age‐related decline in four genotype groups (Ctrl, rod‐*Bmal1KO*, P23H Rho and DM; double mutants combining rod‐*Bmal1KO* with P23H Rho mutations) at four age time points: postnatal day P40, 80, 112, and 180. We found a genotype‐specific effect for both a‐ and b‐wave amplitudes at all ages (Figure 1, Table S2). ERG amplitudes in the rod‐*Bmal1KO* did not show any difference with the Ctrl mice at any of the ages (Figure 1A–H, Table S2). This result suggests that deletion of *Bmal1* in rods up to P180 does not induce any decrease in scotopic and mesopic visual function at midday, as suggested by our previous results.^11^ In comparison with the Ctrl and rod‐*Bmal1KO* groups, significantly decreased a‐ and b‐wave amplitudes were observed in the P23H Rho and the DM mice, already at P40, and they decline throughout the study until P180 (*p* < .05 Holm‐Sidak's post hoc test; Figure 1A–H, Table S2). Importantly, in the P80 and P112 DM mice, the scotopic b‐wave amplitudes were significantly lower than in single P23H Rho mutants (respectively *p* = .0476 and *p* = .0081, Holm‐Sidak's post hoc test; Figure 1F,G, Table S2). In addition, direct comparison of ERG data from P23H Rho and DM groups showed a significantly reduced a‐wave amplitude in the DM at P40, but this difference was not retained at later ages (genotype effect, *p* = .013, Mixed‐effects model analysis; Figure 1A, Table S3). Taken together, these results suggest that the combination of *Bmal1* KO with the P23H mutation of rhodopsin enhanced photoreceptor dysfunction, with rod function being affected as early as P40. They also suggest that this effect translates into accelerated dysfunction of the whole retina, as indicated by reduced b‐wave amplitudes in DM vs. P23H Rho at both P80 and P112. Finally, at P180, the a‐ and b‐waves in DM and P23H Rho mice were similar, with a‐waves being nearly undetectable (Figure 1D,H, Tables S2, S3). This indicates that the light response was mainly lost in both genotype groups at this age. Analysis of oscillatory potentials across all age groups (Figure 1I–L) leads to similar conclusions. Indeed, a significant reduction in the amplitude of OPs occurs already at P40 in the DM light response and progressively decreases until P180 when they are almost undetectable, likely reflecting the gradual decay in the input from degenerating photoreceptors to the inner retina. As for the a‐ and b‐wave amplitudes, at P180, the OP signal from the P23H Rho mutants is mostly absent as well (Figure 1L). To further define the impact of these mutations on retinal function, we also explored photopic light responses in the four genotype groups at P80, P112, and P180 (Figure S4). Our results confirmed that the retina in DM mice was the most dramatically altered, with significantly reduced b‐wave amplitudes when compared to Ctrl and rod‐*Bmal1KO* groups (genotype effect *p* = .0002; Tukey's post hoc test *p* < .01; Table S4).

### 3.2 Histological examination at P45 confirms early photoreceptor alteration in DM mice

**FIGURE 2:**
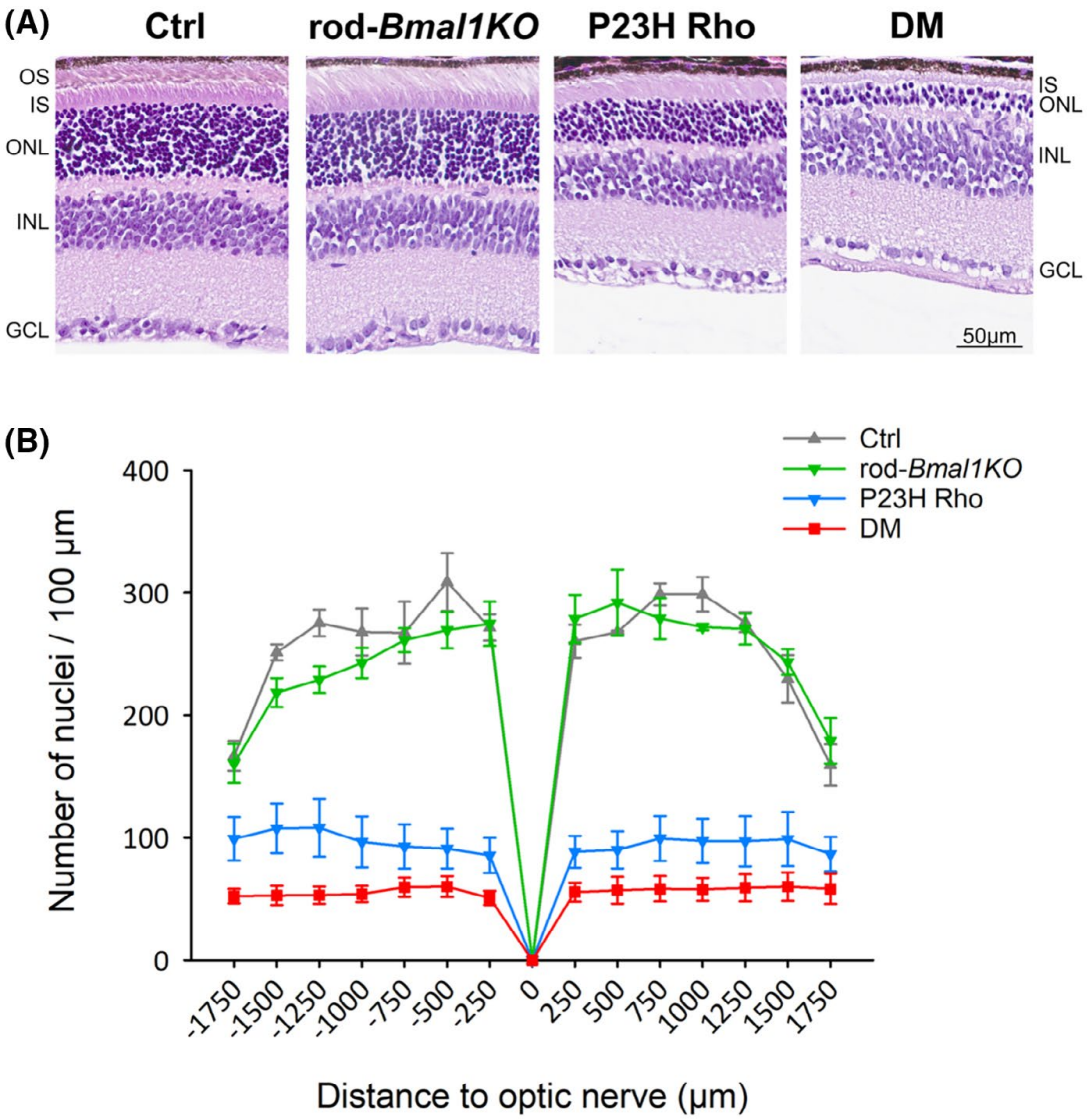
Alteration of the photoreceptor layer in the DM retina at P45. (A) Representative images of retinal sections stained with H&E from DM mice and their distinct controls (Ctrl, rod‐*Bmal1KO*, and P23H Rho) at P45. No abnormalities were observed in Ctrl and rod‐*Bmal1KO* retinas. Loss of photoreceptor cells was visible in the P23H Rho retina sections and a more marked loss was observed in DM mice. OS, outer segments; IS, inner segments; ONL, outer nuclear layer; INL, inner nuclear layer; GCL, ganglion cell layer. (B) Morphometric quantification of histological retinal sections from Ctrl (gray), rod‐*Bmal1KO* (green), P23H Rho (blue), and DM (red) mice: Photoreceptor nuclei were counted within a 100 μm‐width rectangle displaced along the retina, on both sides of the optic nerve head. A significant reduction in number of photoreceptor nuclei in both P23H Rho and DM mice was observed, compared with either Ctrl or rod‐*Bmal1KO* retinas (Two‐way repeated measures ANOVA, Holm‐Sidak's post hoc test; *p* < .001). Data are presented as mean ± SEM; *n* = 3–4 mice/genotype. Scale bars: 50 μm.

Basing on the alterations observed in light responses at P40 in DM mice, we performed a histological examination at a similar early age (P45) by H&E staining of transversal eye sections collected from the four genotype groups (Figure 2). Retinas from Ctrl and rod‐*Bmal1KO* mice showed normal morphology (Figure 2A). By contrast, the outer nuclear layer (ONL) was reduced in P23H Rho, and this decrease appeared more marked in DM retinas (Figure 2A), as confirmed by quantification of photoreceptor nuclei (Two‐way repeated measures ANOVA: genotype effect *p* < .001; Holm‐Sidak's post hoc test *p* < .001 for the comparison between Ctrl and rod‐*Bmal1KO* on one hand and P23H Rho and DM on the other; Figure 2B). However, the reduction of the ONL in the DM eyes did not reach, but was close to, statistical significance when compared to the P23H Rho group (*p* = .067). There was also a genotype‐specific effect on the length of the inner + outer segments measured at 750 μm from the optic nerve head—in particular, outer segments were mostly absent in DM—and the thickness of outer plexiform layers (One‐way ANOVA, *p* < .001 and *p* = .009 respectively; Figure S5). By contrast, there was no genotype effect on the cell density and/or thickness of the inner nuclear layer, the inner plexiform layer, and the ganglion cell layer (One‐way ANOVA, *p* > .05; Figure S5). These results suggest that the disruption of *Bmal1* in rods of P23H Rho mice accelerates photoreceptor degeneration as early as P45.

### 3.3 Immunohistological examination confirms that *Bmal1* disruption in rods promotes rod and cone loss induced by the P23H mutation

**FIGURE 3:**
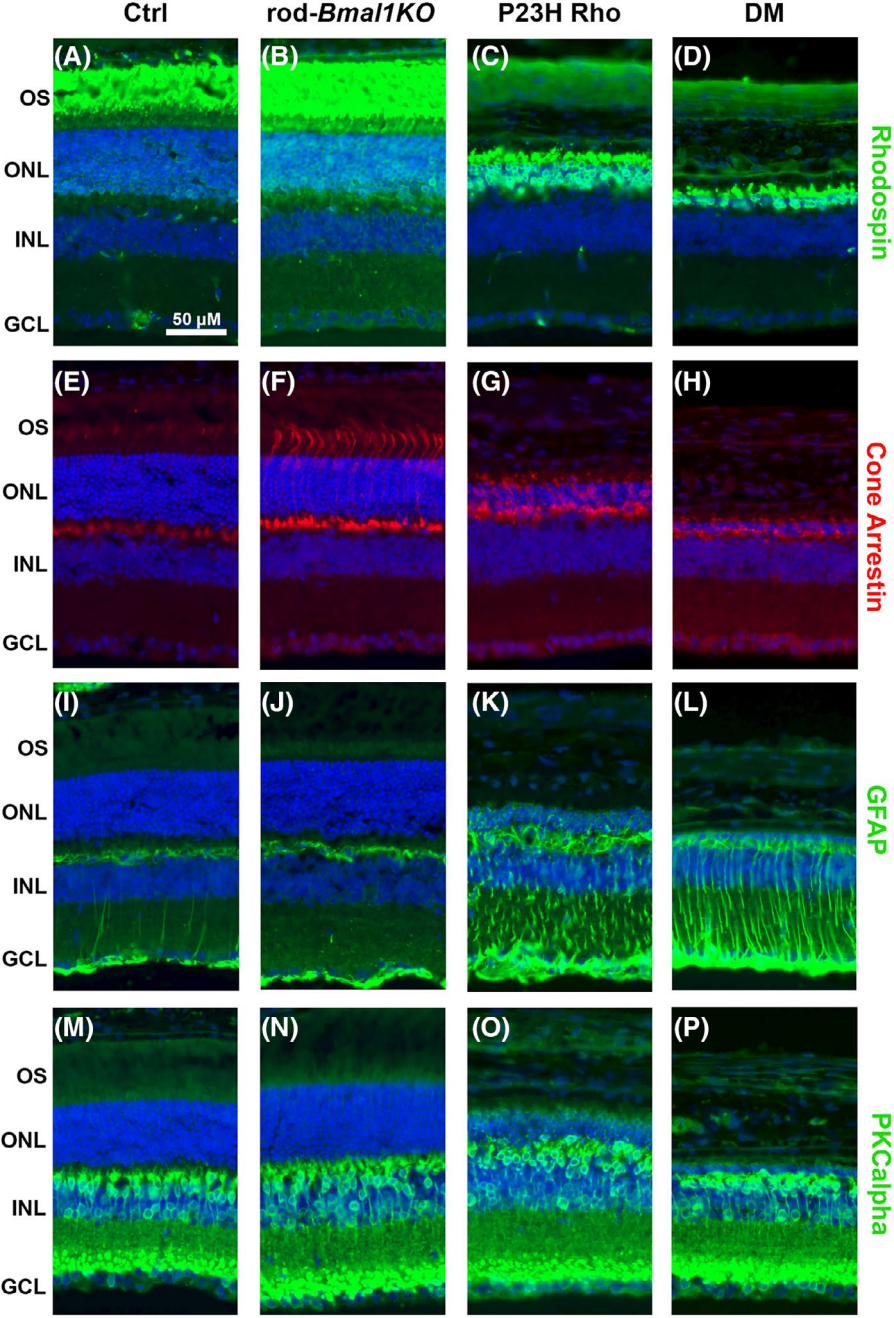
DM retinas show enhanced degenerative process. Representative images of immunohistochemical staining from Ctrl (A, E, I, M), rod‐*Bmal1KO* (B, F, J, N), P23H Rho (C, G, K, O), and DM (D, H, L, P) P120 retina sections (*n* = 4 per genotype). Samples were stained for: Rhodopsin (A–D), cone arrestin (E–H), GFAP (I–L), PKCα (M–P). Rhodopsin is expressed in the photoreceptor outer segments in Ctrl (A) and rod‐*Bmal1KO* (B) mice. Rhodopsin expression is decreased in P23H Rho retinas (C) and mostly disappeared in DM retinas (D). The cone photoreceptor staining with cone arrestin was severely reduced in P23H Rho retina (G) and almost lost in DM retina (H), in which only few cells were labeled at synaptic terminals. P23H Rho and DM retinas showed activated Müller glial cells, detected by increased level of GFAP (K, L). The dendritic processes of bipolar cells (labeled with anti‐PKCα) also appeared affected in these genotype groups (O, P). DAPI staining is shown in blue. GCL, ganglion cell layer; INL, inner nuclear layer; ONL, outer nuclear layer; OS, outer segments. Scale bar: 50 μm.

Further structural examination of retinas was performed at P120, by immunohistochemistry. We first performed immunostaining of retinal sections for the rhodopsin protein. Ctrl and rod‐*Bmal1KO* mice displayed intense labeling of rhodopsin, localized to the photoreceptor outer segments (Figure 3A,B). In contrast, staining of P23H Rho retinas confirmed the reduced presence of rod photoreceptors and showed that rhodopsin was mis‐localized to the inner segments and cell bodies (Figure 3C). Importantly, DM retinas presented a more severe pathological picture, with complete absence of outer segments (as already observed at P45) and only a few stained cell bodies and inner segments (Figure 3D).

**FIGURE 4:**
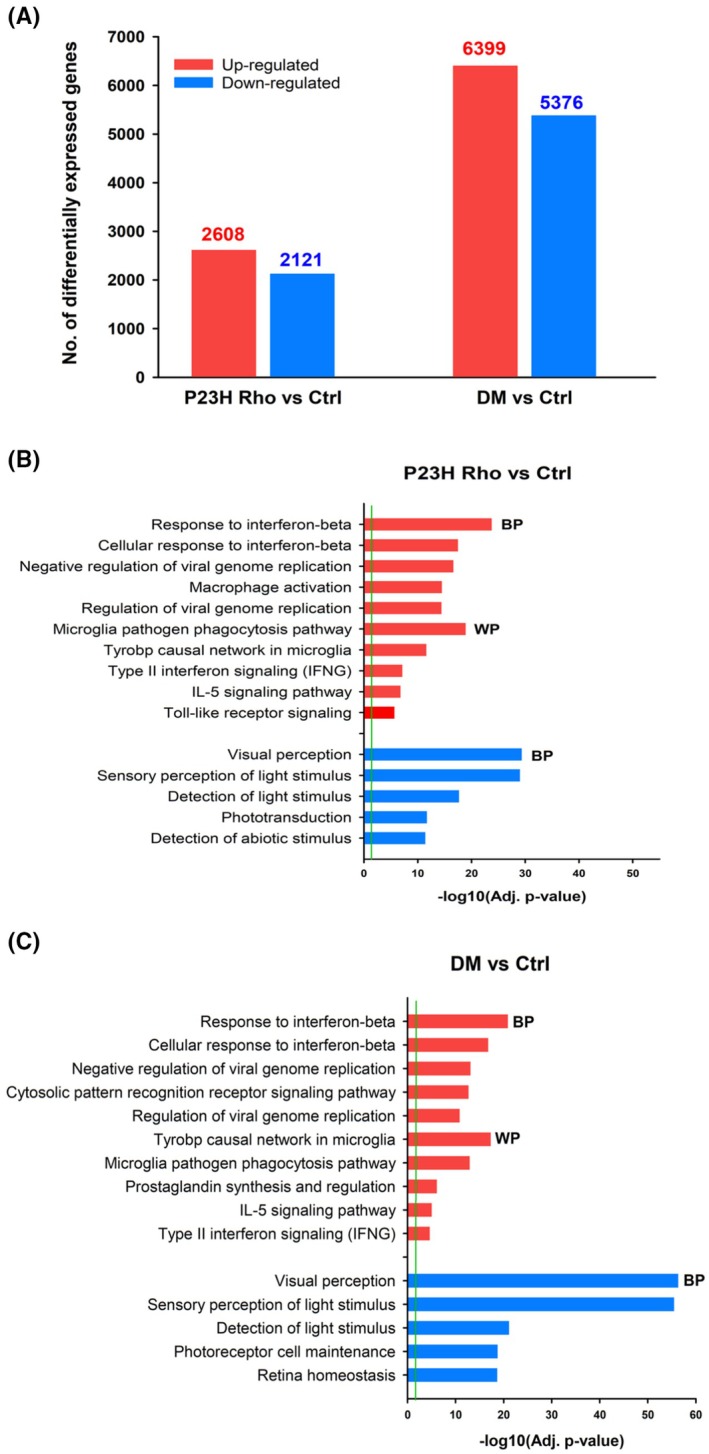
Transcriptional profiling of P23H Rho single mutant and DM double mutant mouse retinas with respect to Ctrl at P120 shows extensive alteration of gene expression in DM. (A) Histogram shows differentially expressed genes in P23H Rho vs. Ctrl and DM vs. Ctrl (Adj. *p* < .05) mouse retinas (up‐regulated and down‐regulated; marked in red, and blue, respectively). (B, C) Functional annotation using gProfiler of differentially expressed genes (DEG) (up‐regulated in red, down‐regulated in blue) in P23H Rho vs. Ctrl (B) and DM vs. Ctrl (C). The 5 most significant biological pathways from the Gene Ontology Biological processes (BP) and (when relevant) WikiPathways (WP) databases are shown. Detailed lists of DEG in P23H Rho vs. Ctrl and DM vs. Ctrl with additional cut offs of fold change ≤ −2 or ≥2 are shown in Tables S5 and S6 respectively. Green line: Significance level cut off (Adj. *p* = .05).

To investigate whether cones were also affected at this stage, we used anti‐cone arrestin labelling. While controls showed the expected labelling of cone pedicles and outer segments (Figure 4E,F), staining of the aforementioned structures was mostly absent in P23H Rho sections (Figure 3G). In DM retinas, the staining was further reduced; no cone outer segment was visible anymore (Figure 4H). This is in agreement with photopic ERG data that showed the absence of light response at P112 (Figure S4). Glial fibrillary acidic protein (GFAP, a glial cell marker) immunoreactivity confirmed the retinal pathology in these P23H Rho and DM genotype groups, with intense staining reflecting reactive gliosis in Müller cells (Figure 3K,L); while the retinas of the Ctrl and rod‐*Bmal1KO* did not show excessive GFAP staining (Figure 3I,J).^58^

Finally, we investigated the structural integrity of the rod bipolar cells by immunostaining against protein kinase C alpha (PKCα). In the Ctrl and rod‐*Bmal1KO* retinas, rod bipolar cells were found in the inner nuclear layer, as expected, and presented normal dendritic processes in the outer plexiform layer (Figure 3M,N). In the P23H Rho and DM retinas, bipolar cells were localized at the correct layer, but their dendritic processes in the outer plexiform layer were of reduced length or totally absent (Figure 3O,P).

### 3.4 *Bmal1* gene disruption in rods exacerbates the transcriptional response to the P23H rhodopsin mutation

To get insight into the molecular mechanisms underlying the observed structural and functional retinal degeneration differences between our mice models, we performed RNA‐sequencing on whole retinas from P120‐aged mice (sampled at midday: ZT6, with ZT0 being the time of lights on). First, we compared the retinal transcriptomes of the P23H Rho and DM genotype groups to our wildtype controls (Ctrl group). We found 4729 differentially expressed genes (DEG) in retinas from P23H Rho relative to the Ctrl group, with 2608 upregulated and 2121 downregulated genes (Adj. *p* < .05) (Figure 4A). By contrast, there were many more (11775) DEG when comparing DM to Ctrl, with 6399 upregulated and 5376 downregulated genes (Figure 4A). These data confirm that, on a molecular level, the combination of the P23H rhodopsin mutation with *Bmal1* disruption induces substantially more profound alterations in the retina than the P23H single mutation alone. RT‐qPCR on RNA samples extracted from the same genotype groups was performed for a few DEG and validated the RNA‐seq data. In particular, rhodopsin gene expression was found drastically decreased with respect to Ctrl in the simple P23H Rho mutant (around 70%) and even more in the DM (93%) retinas, also in agreement with immunohistochemistry data (Figure S6).

We performed functional enrichment analysis to identify significantly altered processes and biological pathways in these transcriptome comparisons. To get more specific insight into the occurring events, we performed this analysis on a subset of DEG that show at least two‐fold changes (up or down) with respect to Ctrl (Tables S5 and S6). Our data showed that genes with increased expression (625 genes) in P23H Rho vs. Ctrl were involved in the immune system, defense response, and glial cell activation (Figure 4B, Table S7). As expected based on our immunohistochemistry results, genes whose expression was decreased (337) were enriched in vision‐related biological processes from the GO database, such as “Visual perception” and “Phototransduction” (Figure 4B, Table S7). Analysis of genes found differentially expressed between DM and Ctrl groups similarly showed marked enrichment in pathways related to immune defense response and glial activation among upregulated transcripts (1843 genes) (Figure 4C, Table S8). Expectedly, GO analysis revealed highly significant gene clusters associated with vision among the 1367 genes with decreased relative expression (Figure 4C, Table S8).

**FIGURE 5:**
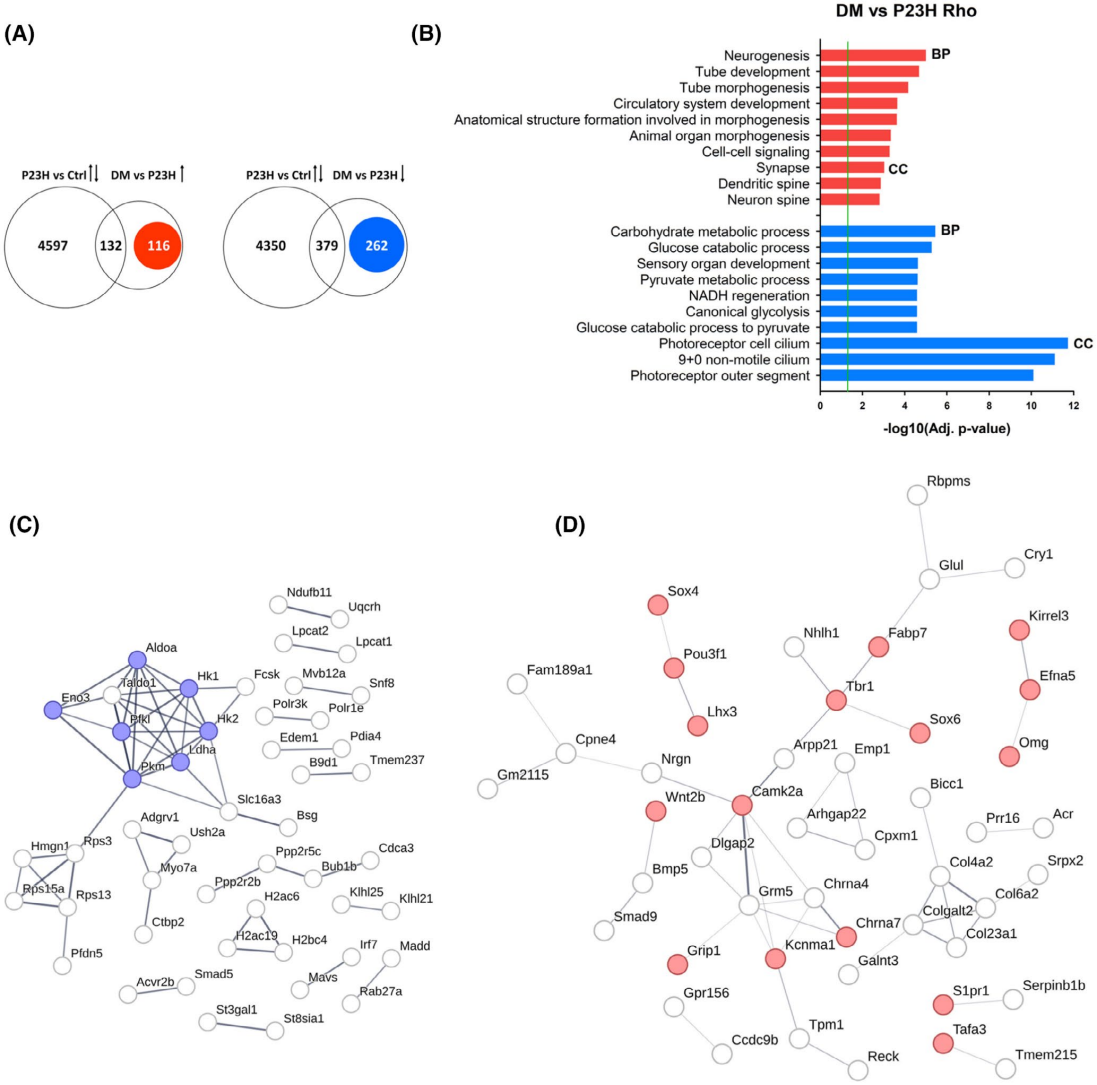
Analysis of uniquely differentially expressed genes between DM and P23H Rho retinas. (A) Venn diagram showing the overlap between genes that are significantly downregulated or upregulated in DM retinas as compared to P23H Rho retinas and differentially expressed between P23H Rho and Ctrl retinas, in P120 mice (*n* = 4/genotype). Numbers of DEG specifically between DM and P23H Rho are highlighted (red, up; blue, down). (B) Most significant biological pathways from the Gene Ontology Biological processes (BP) and Cellular component (CC) categories for genes that are significantly up (116) and downregulated (262) in DM retinas vs. P23H Rho (red, up; blue, down) (detailed lists of pathways in Table S11). (C, D) STRING network analysis of functional associations (edges) of protein products (nodes) for gene lists highlighted in (A). The figure specifically highlights the protein network related to the Glycolysis/Neoglucogenesis KEGG pathway (blue nodes) among the 262 downregulated genes (C; interaction confidence score 0.7) and to the Neurogenesis BP (red nodes) among the 116 upregulated genes (D; interaction confidence score 0.3).

To better understand which molecular changes are specifically occurring in DM retinas, we next compared the transcriptomes between the DM and P23H Rho samples. We found 889 DEG, with 248 upregulated and 641 downregulated (Adj. *p* < .05) (Table S9). Based on Venn diagram analysis, among the 248 upregulated genes, 132 also varied in P23H Rho vs. Ctrl; and among the 641 downregulated genes, 379 also were DEG in P23H Rho vs. Ctrl (Figure 5A). Since functional enrichment did not show major differences between P23H Rho and DM retinas (Figure 4B,C), we performed pathway analysis on the genes that vary specifically in the DM vs. P23H Rho comparison but that do not vary in the P23H Rho vs. Ctrl: hence 116 genes that are up‐ and 262 genes that are downregulated (Figure 5A and Table S10). As seen in Figure 5B, the 262 DEG specifically downregulated in the DM were enriched in Biological processes related to metabolism (i.e. “Carbohydrate metabolic process”, “NADH regeneration”: for instance *Eno3, Hk1, Pkm*) and, expectedly, also to eye development (“Photoreceptor cilium” and “Photoreceptor outer segment” Cellular compartment GO terms with genes such as *Prom1, Mak, Rd3*) (Table S11). We used STRING analysis to identify protein interactions in which products of the same 262 genes might be involved. This analysis revealed a network of 60 edges (Protein–protein interaction enrichment *p* = .000269) and functional associations such as a cluster of seven gene products (*Pfkfb2*, *Pfkl*, *Pkm*, *Aldoa*, *Eno3*, *Hk1*, *Ldha*) that are associated with the “Glycolysis/Gluconeogenesis” pathway (Figure 5C). This is likely the signature of exacerbated loss of the highly metabolically active photoreceptor cells in DM compared to P23H Rho retinas.

By contrast, the 116 upregulated DEG in DM vs. P23H Rho were enriched in Biological processes and Cellular compartments related to neural development such as “Neurogenesis” and “Synapse” (i.e. *Grip1*, *Efna5*, *Camk2a*) (Figure 5B; Table S11). STRING analysis of products of the same 116 genes (48 edges, Protein–protein interaction enrichment *p* = 1.37 × 10^−9^) confirmed enrichment in factors linked to “Neurogenesis” and showed that they interact within a network including *Fabp7, Tbr1, Sox6, Camk2a, Grm5, Chrna7, Kcnma1, Grip1*, among others (Figure 5D). Taken together, these data suggest that activation of developmental pathways and tissue reorganization, which are specific to late stages of retinal degeneration,^59^ occur in DM retinas. To further investigate which mechanisms might trigger these phenotypic changes specifically in the DM, we examined the regulatory (promoter, enhancer, CTCF‐binding) regions of this group of 116 DM vs. P23H Rho upregulated genes by concentrating on evolutionarily conserved sequences. We found highly significant enrichment in binding sites for transcription factors involved in retina differentiation and neural cell fate such as *NeuroD2*,^60^ confirming that retinal developmental mechanisms are re‐activated in the P120‐aged DM (Table S12).

### 3.5 Identification of potential *Bmal1* targets from differential RNAseq analysis

To identify potential genes/pathways that might underlie the synergy between *Bmal1* invalidation and rod degeneration, we finally compared the transcriptomes from rod‐*Bmal1KO* and *Bmal1* ^*fl/+*^ retinas, also at P120 (Table S13). We found significant differences in expression levels of 23 genes at this unique time point (midday‐ZT6): 10 genes up and 13 genes down. As expected, these include clock genes such as *Cry1* and *Nfil3* (up) and *Per3* and *Nr1d2* (down) but also a few other genes, notably linked to retinal degeneration such as *Myo7A* ^61^ that was substantially downregulated, or *Loxl4* and *Ppard* that were highly upregulated.

## 4 DISCUSSION

In this study, we investigated the molecular and pathophysiological effects of combining circadian clock dysfunction and retinal degeneration, most importantly rhodopsin‐linked RP. On molecular, morphological, and functional levels, our data support the hypothesis that a dysfunctional circadian clock can, cell‐autonomously, exacerbate degeneration in a retinal genetic disease. In summary, we found that mice with conditional, rod‐specific *Bmal1* knockout display retinal light responses and photoreceptor integrity similar to their Ctrl littermates. Mice (P23H Rho) with P23H‐induced disease show a typical RP phenotype with visual function decline already at P40, consistent with earlier studies including ours.^34^, ^41^, ^62^ Most importantly, the rod‐specific invalidation of *Bmal1* in these mice (DM) exacerbates these phenotypes. Furthermore, transcriptome analysis shows major gene expression changes between DM and P23H Rho retinas that are linked to regulation of retina differentiation and tissue remodeling in the case of up‐regulated genes, indicating a more advanced stage of degeneration. Genes whose expression is decreased, besides coding for phototransduction proteins, showed marked enrichment in metabolic pathways. Together, these data suggest that pathways controlled by the circadian clock in rods are protective and support functional adaptation in response to genetic retinal disease.

There are a number of similarities between our functional analysis and those reported in the literature. Globally, our results based on the (heterozygous) P23H Rho model are in agreement with those of the literature.^41^, ^62^ Indeed, we found a significant (>50%) reduction of the scotopic a‐wave amplitude in P23H Rho compared with Ctrl mice at P40 (Figure 1). In contrast, there are also differences: for example, between our comparative transcriptome analysis and those reported in the literature. Leinonen and co‐workers described a transcriptomic fingerprint of neuronal network adaptation following early retinal degeneration already detectable at postnatal age 1 month in P23H Rho retinas when compared to their age‐matched littermate wild types.^62^ Unexpectedly, we did not detect any such changes between P23H Rho and Ctrl retinas, albeit at postnatal 4 months. We found more immune response and cell stress‐associated GO terms and pathways (Table S7). This might be due to the more stringent parameters chosen for our RNA‐seq data analysis (FC ≤‐2 or ≥2), which yields more specific results than the previous study. Regarding clinical data, residual light response in RP patients has been proposed to involve cones.^63^ Accordingly, we found no loss in cone function, as measured by light‐adapted ERG in P23H Rho mice. It appeared, however, decreased at 6 months when compared to the Ctrl group (Figure S4).

A number of previous studies reported the effects of clock disruption on retinal phenotype. In the present study, we found that the rod‐mediated visual response measured during daytime did not show any major disturbances in the rod‐*Bmal1KO* mice in the early months of life (Figure 1). These retinas showed neither any obvious loss of photoreceptor cells nor any stress response upon immunostaining or RNA sequencing at 4 months (Figure 3 and Table S13). We recently showed that these rod‐*Bmal1KO* mice did not differ from controls in rhodopsin mRNA expression levels at 4 months of age.^11^ In agreement with these observations, mice with global deletion of *Bmal1* were reported to retain normal retinas up to 6 months.^26^, ^64^ More generally, deletion of core clock components does not lead to drastic alterations of retinal anatomy in young animals.^43^, ^64^, ^65^ However, there is evidence that photoreceptor viability decreases in *Bmal1* or *Clock/Npas2* mutant mice aged 8 months.^28^ The effects of retina‐specific *Bmal1* deletion indicate this is specifically affecting cones, with minor effects on the rod pathway (stunted bipolar cell dendrites, reduced scotopic b‐waves amplitude).^27^ Taken together, these data indicate that clock dysfunction has a mild impact on retinas, except during aging. Nonetheless, in the present study, when comparing the transcriptomes from DM and P23H Rho retinas, we did find pathways such as Neurogenesis, Synapse, and Dendritic spine to be highly significant in up‐regulated genes. Thus, in the case of a pathological state, *Bmal1* invalidation in rods obviously impels the degenerating retina to engage in extensive reorganization of the cellular network. In support of this conclusion, we confirmed activation of genes (*Lhx3* and *Efna5*) respectively involved in bipolar cell development and axon pathfinding,^66^, ^67^ processes that are activated upon cellular reprogramming during advanced RP.^59^

How then does clock disturbance in rods aggravate rod degeneration? Our longitudinal ERG study indicates a synergistic effect of clock disruption and retinal degeneration that is already significant at P40 in rods. This is corroborated by the transcriptome analysis performed at 4 months and showing more severe loss of photoreceptor and phototransduction‐related genes in DM vs. P23H Rho samples. Data from the literature showed that the circadian clock plays a broad role in maintaining health during aging. Indeed, mice deficient in *Bmal1* display reduced lifespan and symptoms of premature aging, including cataracts and neurodegeneration.^68^, ^69^ Similarly, circadian disruption induced by exposure to chronic jet lag reduces lifespan and alters brain activity in animal models.^70^, ^71^ In humans, circadian misalignment or clock (*BMAL1*) gene polymorphism has been associated with the occurrence of neurodegenerative diseases such as Alzheimer's and Parkinson's disease.^72^, ^73^, ^74^ The link between clock gene mutation and neuronal pathology in mice involves impairment of redox homeostasis.^69^ Oxidative and reductive cycles synchronize to 24‐h rhythms through tight regulation of metabolism by circadian clocks, at least in the periphery, but this relationship has not been clearly demonstrated in the central nervous system (reviewed by Ref. [75]). In peripheral cells, a strong reciprocal link exists between clock factors and metabolic processes, at several levels. Among these, NAD(P)(H) is at the intersection between metabolism, circadian rhythms, and aging.^76^ Also, mitochondrial metabolic activity and nutrient use are under circadian control.^77^, ^78^ Our analysis of DM vs. P23H Rho downregulated genes showed highly significant enrichment in metabolic pathways such as glycolysis and NADH regeneration, either indicating that these processes might be particularly dysfunctional in the DM or reflecting the dramatic loss of photoreceptor cells. Thus, one hypothesis is that *Bmal1* might act through the loss of homeostatic mechanisms such as regulation of metabolism and of oxidative stress, that play crucial roles in rods.^79^ In support of that, key metabolism regulators such as *Sirt1* and *Pgc‐1α* display daily mRNA expression rhythms in the retina.^80^, ^81^ Finally, circadian time‐affected genes in mouse photoreceptors are enriched in biological pathways related to metabolism.^19^ Despite the accumulating evidence, the exact BMAL1 metabolic targets involved in the retinal phenotype of the DM remain presently to be identified.

Given that rod‐*Bmal1KO* retinas do not show obvious signs of retinal disease at 4 months, the identity of the genes that are up‐ or downregulated in this mutant with respect to the *Bmal* ^*fl/+*^ control might also provide some hint of the molecular changes that underlie the synergy with P23H Rho effects. For instance, we detected a dramatic decrease (18‐fold) in expression of the transcript encoding Myosin VIIa (Myo7a), a constituent of the photoreceptor connecting cilium^82^ considered to play a role in rhodopsin transport to the outer segment.^83^ Human Myosin VIIa mutations cause Usher1B vision/hearing loss.^84^ Although loss or mutation of this gene in mice could not be phenotypically correlated with retinal degeneration,^61^, ^85^ it is tempting to speculate that in the context of P23H Rho, suboptimal transport of outer segment constituents due to the absence of Myosin VIIa might further weaken photoreceptor structure and precipitate their death.

There are limitations in our study. In particular, the molecular data, although providing strong support to our hypothesis, did not disclose the mechanisms of synergy between *Bmal1* invalidation and RP. Insight into this interaction might require single cell sequencing or similar analyses at earlier stages of the disease. The strength of our study is the use of a combination of well‐characterized mouse models together with functional and molecular approaches to understand the interaction between clock dysfunction and rod disease. Our data support the existence of a circadian clock in rods that not only controls their daily light response but also confers a neuroprotective role. Moreover, these results confirm similar data obtained in another model. Indeed, Jauregui‐Lozano and co‐workers showed the critical role of the CLOCK:CYCLE complex in counteracting oxidative stress and ensuring photoreceptor homeostasis upon aging in *Drosophila*.^86^ Thus, our study, taken together with other reports, highlights the importance of a functional clock in photoreceptor health, not only in aging pathology but also in the early steps of severe, early onset genetic disease such as RP. The double mutant model generated here likely constitutes a valuable, innovative experimental model to understand these early protective mechanisms of the circadian clock and clock genes in photoreceptors.

## AUTHOR CONTRIBUTIONS

S.T.G. performed experiments, analysis, prepared figures, and wrote the manuscript. A.B., P.D.M., and A.J. performed bioinformatics analysis and edited the manuscript. C.S. provided technical assistance and edited the manuscript. J.B.t.B. provided technical assistance. N.M. analyzed data and edited the manuscript. A.A.B. and M.‐P.F.‐S. conceptualized and directed the project, obtained funding, provided resources, performed analysis, and edited the manuscript.

## DISCLOSURES

The authors declare no competing interests.

## Supporting information


Figures S1‐S6:

Tables S1‐S13:

## Data Availability

The data supporting the findings of this study are available in the Results and Supplemental Material of this article.

## References

[1] Koronowski KB , Sassone‐Corsi P . Communicating clocks shape circadian homeostasis. Science. 2021;371:eabd0951. doi:10.1126/science.abd0951 33574181 PMC8123919

[2] Takahashi JS , Hong HK , Ko CH , McDearmon EL . The genetics of mammalian circadian order and disorder: implications for physiology and disease. Nat Rev Genet. 2008;9(10):764‐775. doi:10.1038/nrg2430 18802415 PMC3758473

[3] Zhang R , Lahens NF , Ballance HI , Hughes ME , Hogenesch JB . A circadian gene expression atlas in mammals: implications for biology and medicine. Proc Natl Acad Sci USA. 2014;111:16219‐16224. 25349387 10.1073/pnas.1408886111 PMC4234565

[4] Chaix A , Zarrinpar A , Panda S . The circadian coordination of cell biology. J Cell Biol. 2016;215:15‐25. 27738003 10.1083/jcb.201603076 PMC5057284

[5] Mure LS , Le HD , Benegiamo G , et al. Diurnal transcriptome atlas of a primate across major neural and peripheral tissues. Science. 2018;359(6381):eaao0318. doi:10.1126/science.aao0318 29439024 PMC5924732

[6] West AC , Bechtold DA . The cost of circadian desynchrony: evidence, insights and open questions. BioEssays. 2015;37:777‐788. 26010005 10.1002/bies.201400173 PMC4973832

[7] Felder‐Schmittbuhl M‐P , Calligaro H , Dkhissi‐Benyahya O . The retinal clock in mammals: role in health and disease. ChronoPhysiology Ther. 2017;7:33‐45.

[8] McMahon DG , Iuvone PM , Tosini G . Circadian organization of the mammalian retina: from gene regulation to physiology and diseases. Prog Retin Eye Res. 2014;39:58‐76. doi:10.1016/j.preteyeres.2013.12.001 24333669 PMC3927986

[9] Dkhissi‐Benyahya O , Coutanson C , Knoblauch K , et al. The absence of melanopsin alters retinal clock function and dopamine regulation by light. Cell Mol Life Sci. 2013;70:3435‐3447. 23604021 10.1007/s00018-013-1338-9 PMC11113582

[10] Jaeger C , Sandu C , Malan A , Mellac K , Hicks D , Felder‐Schmittbuhl MP . Circadian organization of the rodent retina involves strongly coupled, layer‐specific oscillators. FASEB J. 2015;29:1493‐1504. 25573753 10.1096/fj.14-261214

[11] Gegnaw ST , Sandu C , Mendoza J , Bergen AA , Felder‐Schmittbuhl MP . Dark‐adapted light response in mice is regulated by a circadian clock located in rod photoreceptors. Exp Eye Res. 2021;213:108807. 34695438 10.1016/j.exer.2021.108807

[12] Liu X , Zhang Z , Ribelayga CP . Heterogeneous expression of the core circadian clock proteins among neuronal cell types in mouse retina. PLoS One. 2012;7:e50602. 23189207 10.1371/journal.pone.0050602 PMC3506613

[13] Ruan GX , Zhang DQ , Zhou T , Yamazaki S , McMahon DG . Circadian organization of the mammalian retina. Proc Natl Acad Sci USA. 2006;103:9703‐9708. 16766660 10.1073/pnas.0601940103 PMC1480470

[14] Bagchi U , Gegnaw ST , Milićević N , et al. Core‐clock genes period 1 and 2 regulate visual cascade and cell cycle components during mouse eye development. Biochim Biophys Acta Gene Regul Mech. 2020;1863:194623. 32795630 10.1016/j.bbagrm.2020.194623

[15] Sawant OB , Horton AM , Zucaro OF , et al. The circadian clock gene Bmal1 controls thyroid hormone‐mediated spectral identity and cone photoreceptor function. Cell Rep. 2017;21(3):692‐706. doi:10.1016/j.celrep.2017.09.069 29045837 PMC5647869

[16] Bobu C , Hicks D . Regulation of retinal photoreceptor phagocytosis in a diurnal mammal by circadian clocks and ambient lighting. Invest Ophthalmol Vis Sci. 2009;50(7):3495‐3502. doi:10.1167/iovs.08-3145 19234351

[17] DeVera C , Dixon J , Chrenek MA , et al. The circadian clock in the retinal pigment epithelium controls the diurnal rhythm of phagocytic activity. Int J Mol Sci. 2022;23:5302. 35628111 10.3390/ijms23105302 PMC9141420

[18] Krigel A , Felder‐Schmittbuhl MP , Hicks D . Circadian‐clock driven cone‐like photoreceptor phagocytosis in the neural retina leucine zipper gene knockout mouse. Mol Vis. 2010;16:2873‐2881. 21203345 PMC3013069

[19] Milićević N , Ait‐Hmyed Hakkari O , Bagchi U . Core circadian clock genes Per1 and Per2 regulate the rhythm in photoreceptor outer segment phagocytosis. FASEB J. 2021;35(7):e21722. doi:10.1096/fj.202100293RR 34160105

[20] Organisciak DT , Darrow RM , Barsalou L , Kutty RK , Wiggert B . Circadian‐dependent retinal light damage in rats. Invest Ophthalmol Vis Sci. 2000;41:3694‐3701. 11053264

[21] Vaughan DK , Nemke JL , Fliesler SJ , Darrow RM , Organisciak DT . Evidence for a circadian rhythm of susceptibility to retinal light damage. Photochem Photobiol. 2002;75:547‐553. 12017483 10.1562/0031-8655(2002)075<0547:efacro>2.0.co;2

[22] Baba K , Pozdeyev N , Mazzoni F , et al. Melatonin modulates visual function and cell viability in the mouse retina via the MT1 melatonin receptor. Proc Natl Acad Sci USA. 2009;106:15043‐15048. 19706469 10.1073/pnas.0904400106 PMC2736407

[23] Di R , Luo Q , Mathew D , Bhatwadekar AD . Diabetes alters diurnal rhythm of electroretinogram in db/db mice. Yale J Biol Med. 2019;92:155‐167. 31249476 PMC6585529

[24] Barnard AR , Hattar S , Hankins MW , Lucas RJ . Melanopsin regulates visual processing in the mouse retina. Curr Biol. 2006;16:389‐395. 16488873 10.1016/j.cub.2005.12.045

[25] Cameron MA , Barnard AR , Hut RA , et al. Electroretinography of wild‐type and cry mutant mice reveals circadian tuning of photopic and mesopic retinal responses. J Biol Rhythm. 2008;23:489‐501. 10.1177/0748730408325874 19060258

[26] Storch KF , Paz C , Signorovitch J , et al. Intrinsic circadian clock of the mammalian retina: importance for retinal processing of visual information. Cell. 2007;130:730‐741. 17719549 10.1016/j.cell.2007.06.045 PMC2040024

[27] Baba K , Piano I , Lyuboslavsky P , et al. Removal of clock gene Bmal1 from the retina affects retinal development and accelerates cone photoreceptor degeneration during aging. Proc Natl Acad Sci USA. 2018;115:13099‐13104. 30498030 10.1073/pnas.1808137115 PMC6305005

[28] Baba K , Ribelayga CP , Michael Iuvone P , Tosini G . The retinal circadian clock and photoreceptor viability. Adv Exp Med Biol. 2018;1074:345‐350. 29721962 10.1007/978-3-319-75402-4_42 PMC6003627

[29] Ferrari S , Di Iorio E , Barbaro V , Ponzin D , Sorrentino FS , Parmeggiani F . Retinitis pigmentosa: genes and disease mechanisms. Curr Genomics. 2011;12(4):238‐249. doi:10.2174/138920211795860107 22131869 PMC3131731

[30] Verbakel SK , van Huet RAC , Boon CJF , et al. Non‐syndromic retinitis pigmentosa. Prog Retin Eye Res. 2018;66:157‐186. 29597005 10.1016/j.preteyeres.2018.03.005

[31] Dryja TP , McGee TL , Reichel E , et al. A point mutation of the rhodopsin gene in one form of retinitis pigmentosa. Nature. 1990;343:364‐366. 2137202 10.1038/343364a0

[32] Cremers FPM , Boon CJF , Bujakowska K , Zeitz C . Special issue introduction: inherited retinal disease: novel candidate genes, genotype‐phenotype correlations, and inheritance models. Genes (Basel). 2018;9(4):215. doi:10.3390/genes9040215 29659558 PMC5924557

[33] Sakami S , Kolesnikov AV , Kefalov VJ , Palczewski K . P23H opsin knock‐in mice reveal a novel step in retinal rod disc morphogenesis. Hum Mol Genet. 2014;23:1723‐1741. 24214395 10.1093/hmg/ddt561 PMC3943518

[34] Sakami S , Maeda T , Bereta G , et al. Probing mechanisms of photoreceptor degeneration in a new mouse model of the common form of autosomal dominant retinitis pigmentosa due to P23H opsin mutations. J Biol Chem. 2011;286:10551‐10567. 21224384 10.1074/jbc.M110.209759 PMC3060508

[35] Mattapallil MJ , Wawrousek EF , Chan CC , et al. The Rd8 mutation of the Crb1 gene is present in vendor lines of C57BL/6N mice and embryonic stem cells, and confounds ocular induced mutant phenotypes. Invest Ophthalmol Vis Sci. 2012;53:2921‐2927. 22447858 10.1167/iovs.12-9662 PMC3376073

[36] Li S , Chen D , Sauvé Y , McCandless J , Chen YJ , Chen CK . Rhodopsin‐iCre transgenic mouse line for Cre‐mediated rod‐specific gene targeting. Genesis. 2005;41:73‐80. 15682388 10.1002/gene.20097

[37] Bunger MK , Wilsbacher LD , Moran SM , et al. Mop3 is an essential component of the master circadian pacemaker in mammals. Cell. 2000;103:1009‐1017. 11163178 10.1016/s0092-8674(00)00205-1 PMC3779439

[38] Yoo SH , Yamazaki S , Lowrey PL , et al. PERIOD2::LUCIFERASE real‐time reporting of circadian dynamics reveals persistent circadian oscillations in mouse peripheral tissues. Proc Natl Acad Sci USA. 2004;101:5339‐5346. 14963227 10.1073/pnas.0308709101 PMC397382

[39] Ait‐Hmyed Hakkari O , Acar N , Savier E , et al. Rev‐Erbα modulates retinal visual processing and behavioral responses to light. FASEB J. 2016;30:3690‐3701. 27440795 10.1096/fj.201600414R

[40] Tanimoto N , Muehlfriedel RL , Fischer MD , et al. Vision tests in the mouse: functional phenotyping with electroretinography. Front Biosci (Landmark ed). 2009;14:2730‐2737. 19273231 10.2741/3409

[41] Gegnaw ST , Sandu C , Mazzaro N , Mendoza J , Bergen AA , Felder‐Schmittbuhl MP . Enhanced robustness of the mouse retinal circadian clock upon inherited retina degeneration. J Biol Rhythm. 2022;37:567‐574. 10.1177/07487304221112845 35912966

[42] Bankhead P , Loughrey MB , Fernández JA , et al. QuPath: open source software for digital pathology image analysis. Sci Rep. 2017;7(1):16878. doi:10.1038/s41598-017-17204-5 29203879 PMC5715110

[43] Ait‐Hmyed O , Felder‐Schmittbuhl MP , Garcia‐Garrido M , et al. Mice lacking period 1 and period 2 circadian clock genes exhibit blue cone photoreceptor defects. Eur J Neurosci. 2013;37:1048‐1060. 23351077 10.1111/ejn.12103

[44] Hicks D , Molday RS . Differential immunogold‐dextran labeling of bovine and frog rod and cone cells using monoclonal antibodies against bovine rhodopsin. Exp Eye Res. 1986;42:55‐71. 2420630 10.1016/0014-4835(86)90017-5

[45] Zhu X , Li A , Brown B , Weiss ER , Osawa S , Craft CM . Mouse cone arrestin expression pattern: light induced translocation in cone photoreceptors. Mol Vis. 2002;8:462‐471. 12486395

[46] Kim D , Langmead B , Salzberg SL . HISAT: a fast spliced aligner with low memory requirements. Nat Methods. 2015;12(4):357‐360. doi:10.1038/nmeth.3317 25751142 PMC4655817

[47] Anders S , Pyl PT , Huber W . HTSeq—a python framework to work with high‐throughput sequencing data. Bioinformatics. 2015;31(2):166‐169. doi:10.1093/bioinformatics/btu638 25260700 PMC4287950

[48] Robinson MD , Oshlack A . A scaling normalization method for differential expression analysis of RNA‐seq data. Genome Biol. 2010;11:R25. 20196867 10.1186/gb-2010-11-3-r25 PMC2864565

[49] Ritchie ME , Phipson B , Wu D , et al. Limma powers differential expression analyses for RNA‐sequencing and microarray studies. Nucleic Acids Res. 2015;43:e47. 25605792 10.1093/nar/gkv007 PMC4402510

[50] Law CW , Chen Y , Shi W , Smyth GK . Voom: precision weights unlock linear model analysis tools for RNA‐seq read counts. Genome Biol. 2014;15:R29. 24485249 10.1186/gb-2014-15-2-r29 PMC4053721

[51] Reimand J , Kull M , Peterson H , Hansen J , Vilo J . G:profiler—a web‐based toolset for functional profiling of gene lists from large‐scale experiments. Nucleic Acids Res. 2007;35:W193‐W200. 17478515 10.1093/nar/gkm226 PMC1933153

[52] Szklarczyk D , Franceschini A , Wyder S , et al. STRING v10: protein‐protein interaction networks, integrated over the tree of life. Nucleic Acids Res. 2015;43:D447‐D452. 25352553 10.1093/nar/gku1003 PMC4383874

[53] Karolchik D , Hinrichs AS , Furey TS , et al. The UCSC table browser data retrieval tool. Nucleic Acids Res. 2004;32:D493‐D496. 14681465 10.1093/nar/gkh103 PMC308837

[54] Consortium EP . A user's guide to the encyclopedia of DNA elements (ENCODE). PLoS Biol. 2011;9(4):e1001046. doi:10.1371/journal.pbio.1001046 21526222 PMC3079585

[55] Consortium EP . An integrated encyclopedia of DNA elements in the human genome. Nature. 2012;489:57‐74. 22955616 10.1038/nature11247 PMC3439153

[56] Consortium EP , Moore JE , Purcaro MJ , et al. Expanded encyclopaedias of DNA elements in the human and mouse genomes. Nature. 2020;583(7818):699‐710. doi:10.1038/s41586-020-2493-4 32728249 PMC7410828

[57] Leporcq C , Spill Y , Balaramane D , Toussaint C , Weber M , Bardet AF . TFmotifView: a webserver for the visualization of transcription factor motifs in genomic regions. Nucleic Acids Res. 2020;48:W208‐W217. 32324215 10.1093/nar/gkaa252 PMC7319436

[58] Leinonen HO , Bull E , Fu Z . Neural and muller glial adaptation of the retina to photoreceptor degeneration. Neural Regen Res. 2023;18:701‐707. 36204825 10.4103/1673-5374.354511 PMC9700092

[59] Pfeiffer RL , Marc RE , Jones BW . Persistent remodeling and neurodegeneration in late‐stage retinal degeneration. Prog Retin Eye Res. 2020;74:100771. 31356876 10.1016/j.preteyeres.2019.07.004 PMC6982593

[60] Cherry TJ , Wang S , Bormuth I , Schwab M , Olson J , Cepko CL . NeuroD factors regulate cell fate and neurite stratification in the developing retina. J Neurosci. 2011;31:7365‐7379. 21593321 10.1523/JNEUROSCI.2555-10.2011 PMC3135085

[61] Williams DS . Usher syndrome: animal models, retinal function of usher proteins, and prospects for gene therapy. Vis Res. 2008;48:433‐441. 17936325 10.1016/j.visres.2007.08.015 PMC2680226

[62] Leinonen H , Pham NC , Boyd T , Santoso J , Palczewski K . Homeostatic plasticity in the retina is associated with maintenance of night vision during retinal degenerative disease. eLife. 2020;9:e59422. 32960171 10.7554/eLife.59422 PMC7529457

[63] Hartong DT , Berson EL , Dryja TP . Retinitis pigmentosa. Lancet. 2006;368:1795‐1809. 17113430 10.1016/S0140-6736(06)69740-7

[64] Owens L , Buhr E , Tu DC , Lamprecht TL , Lee J , Van Gelder RN . Effect of circadian clock gene mutations on nonvisual photoreception in the mouse. Invest Ophthalmol Vis Sci. 2012;53:454‐460. 22159024 10.1167/iovs.11-8717 PMC3292377

[65] Selby CP , Thompson C , Schmitz TM , Van Gelder RN , Sancar A . Functional redundancy of cryptochromes and classical photoreceptors for nonvisual ocular photoreception in mice. Proc Natl Acad Sci USA. 2000;97:14697‐14702. 11114194 10.1073/pnas.260498597 PMC18981

[66] Davenport RW , Thies E , Zhou R , Nelson PG . Cellular localization of ephrin‐A2, ephrin‐A5, and other functional guidance cues underlies retinotopic development across species. J Neurosci. 1998;18:975‐986. 9437019 10.1523/JNEUROSCI.18-03-00975.1998 PMC6792763

[67] Dong X , Yang H , Zhou X , et al. LIM‐homeodomain transcription factor LHX4 is required for the differentiation of retinal rod bipolar cells and OFF‐cone bipolar subtypes. Cell Rep. 2020;32(11):108144. doi:10.1016/j.celrep.2020.108144 32937137 PMC9245082

[68] Kondratov RV , Kondratova AA , Gorbacheva VY , Vykhovanets OV , Antoch MP . Early aging and age‐related pathologies in mice deficient in BMAL1, the core componentof the circadian clock. Genes Dev. 2006;20:1868‐1873. 16847346 10.1101/gad.1432206 PMC1522083

[69] Musiek ES , Lim MM , Yang G , et al. Circadian clock proteins regulate neuronal redox homeostasis and neurodegeneration. J Clin Invest. 2013;123:5389‐5400. 24270424 10.1172/JCI70317 PMC3859381

[70] Davidson AJ , Sellix MT , Daniel J , Yamazaki S , Menaker M , Block GD . Chronic jet‐lag increases mortality in aged mice. Curr Biol. 2006;16:R914‐R916. 17084685 10.1016/j.cub.2006.09.058 PMC1635966

[71] Gao Q , Khan S , Zhang L . Brain activity and transcriptional profiling in mice under chronic jet lag. Sci Data. 2020;7:361. 33087702 10.1038/s41597-020-00709-6 PMC7578042

[72] Bokenberger K , Sjölander A , Dahl Aslan AK , Karlsson IK , Åkerstedt T , Pedersen NL . Shift work and risk of incident dementia: a study of two population‐based cohorts. Eur J Epidemiol. 2018;33:977‐987. 30076495 10.1007/s10654-018-0430-8 PMC6153510

[73] Chen Q , Peng XD , Huang CQ , Hu XY , Zhang XM . Association between ARNTL (BMAL1) rs2278749 polymorphism T >C and susceptibility to Alzheimer disease in a Chinese population. Genet Mol Res. 2015;14:18515‐18522. 26782499 10.4238/2015.December.23.39

[74] Gu Z , Wang B , Zhang YB , et al. Association of ARNTL and PER1 genes with Parkinson's disease: a case‐control study of Han Chinese. Sci Rep. 2015;5:15891. 26507264 10.1038/srep15891 PMC4623766

[75] Smith SK , Musiek ES . Impact of circadian and diurnal rhythms on cellular metabolic function and neurodegenerative diseases. Int Rev Neurobiol. 2020;154:393‐412. 32739012 10.1016/bs.irn.2020.02.005 PMC9008766

[76] Levine DC , Ramsey KM , Bass J . Circadian NAD(P)(H) cycles in cell metabolism. Semin Cell Dev Biol. 2022;126:15‐26. 34281771 10.1016/j.semcdb.2021.07.008 PMC8761220

[77] Jacobi D , Liu S , Burkewitz K , et al. Hepatic Bmal1 regulates rhythmic mitochondrial dynamics and promotes metabolic fitness. Cell Metab. 2015;22:709‐720. 26365180 10.1016/j.cmet.2015.08.006 PMC4598294

[78] Neufeld‐Cohen A , Robles MS , Aviram R , et al. Circadian control of oscillations in mitochondrial rate‐limiting enzymes and nutrient utilization by PERIOD proteins. Proc Natl Acad Sci USA. 2016;113:E1673‐E1682. 26862173 10.1073/pnas.1519650113 PMC4812734

[79] Newton F , Megaw R . Mechanisms of photoreceptor death in retinitis pigmentosa. Genes (Basel). 2020;11:1120. 32987769 10.3390/genes11101120 PMC7598671

[80] Ban N , Ozawa Y , Inaba T , et al. Light‐dark condition regulates sirtuin mRNA levels in the retina. Exp Gerontol. 2013;48:1212‐1217. 23648587 10.1016/j.exger.2013.04.010

[81] Kunst S , Wolloscheck T , Hölter P , et al. Transcriptional analysis of rat photoreceptor cells reveals daily regulation of genes important for visual signaling and light damage susceptibility. J Neurochem. 2013;124:757‐769. 23145934 10.1111/jnc.12089

[82] Liu X , Vansant G , Udovichenko IP , Wolfrum U , Williams DS . Myosin VIIa, the product of the usher 1B syndrome gene, is concentrated in the connecting cilia of photoreceptor cells. Cell Motil Cytoskeleton. 1997;37:240‐252. 9227854 10.1002/(SICI)1097-0169(1997)37:3<240::AID-CM6>3.0.CO;2-A

[83] Leung M , Steinman J , Li D , et al. The logistical backbone of photoreceptor cell function: complementary mechanisms of dietary vitamin a receptors and rhodopsin transporters. Int J Mol Sci. 2024;25(8):4278. doi:10.3390/ijms25084278 38673863 PMC11050646

[84] Toms M , Pagarkar W , Moosajee M . Usher syndrome: clinical features, molecular genetics and advancing therapeutics. Ther Adv Ophthalmol. 2020;12:2515841420952194. doi:10.1177/2515841420952194 32995707 PMC7502997

[85] Colella P , Sommella A , Marrocco E , et al. Myosin7a deficiency results in reduced retinal activity which is improved by gene therapy. PLoS One. 2013;8(8):e72027. doi:10.1371/journal.pone.0072027 23991031 PMC3753344

[86] Jauregui‐Lozano J , Hall H , Stanhope SC , Bakhle K , Marlin MM , Weake VM . The clock:cycle complex is a major transcriptional regulator of drosophila photoreceptors that protects the eye from retinal degeneration and oxidative stress. PLoS Genet. 2022;18:e1010021. 35100266 10.1371/journal.pgen.1010021 PMC8830735

