# Supplementary material for: Circadian clock disruption promotes retinal photoreceptor degeneration
Source: FASEB J. 2025 Apr 2;39(7):e70507. doi: 10.1096/fj.202401967R (PMC11962828; doi:10.1096/fj.202401967R)
Supplement: Supplementary file 1 — Figures S1‐S6: [file FSB2-39-e70507-s001.pdf]

Circadian Clock Disruption promotes Retinal Photoreceptor Degeneration

Shumet T. Gegnaw[1,2], Cristina Sandu[1], Amandine Bery[1], Jacoline B. ten Brink[2], Nemanja Milićević[3], Aldo Jongejan[4,5,6], Perry D. Moerland[4,5,6], †Arthur A. Bergen[2,7,8,#*], Marie-Paule Felder-Schmittbuhl[1#*]

[1]Centre National de la Recherche Scientifique, Université de Strasbourg, Institut des Neurosciences Cellulaires et Intégratives, F-67000 Strasbourg, France ; shumet.gegnaw@etu.unistra.fr (S.G), sandu@inci-cnrs.unistra.fr (C.S), bery@inci-cnrs.unistra.fr (A.B), feldermp@inci-cnrs.unistra.fr (M-P.F-S)

[2] Department of Human Genetics, Amsterdam University Medical Centers, location AMC, University of Amsterdam, Meibergdreef 9, 1105 AZ, Amsterdam, the Netherlands; j.b.tenbrink@amsterdamumc.nl (J.B), aabergen@amsterdamumc.nl (A.A.B)

[3] Faculty of Medicine and Health Technology, Tampere University, Arvo Ylpön katu 34, 33520 Tampere, Finland; nemanja.milicevic@tuni.fi (N.M)

[4] Amsterdam UMC, University of Amsterdam, Epidemiology and Data Science, Meibergdreef 9, Amsterdam, Netherlands; a.jongejan@amsterdamumc.nl (A.J), p.d.moerland@amsterdamumc.nl (P.D.M)

[5] Amsterdam Public Health, Methodology Amsterdam, The Netherlands

[6] Amsterdam Institute for Immunology and Infectious Diseases, Amsterdam, The Netherlands

[7] Department of Ophthalmology, Amsterdam University Medical Centers, location AMC, University of Amsterdam, Meibergdreef 9, 1105 AZ, Amsterdam, the Netherlands

[8] Emma Centre for Personalized Medicine, Amsterdam University Medical Centers, location AMC, University of Amsterdam, Meibergdreef 9, 1105 AZ, Amsterdam, the Netherlands

#equal last author contribution

†The author passed away on September 15th 2024

***** Correspondence:

Dr. Marie-Paule Felder-Schmittbuhl, Email address: feldermp@inci-cnrs.unistra.fr

Supplementary data

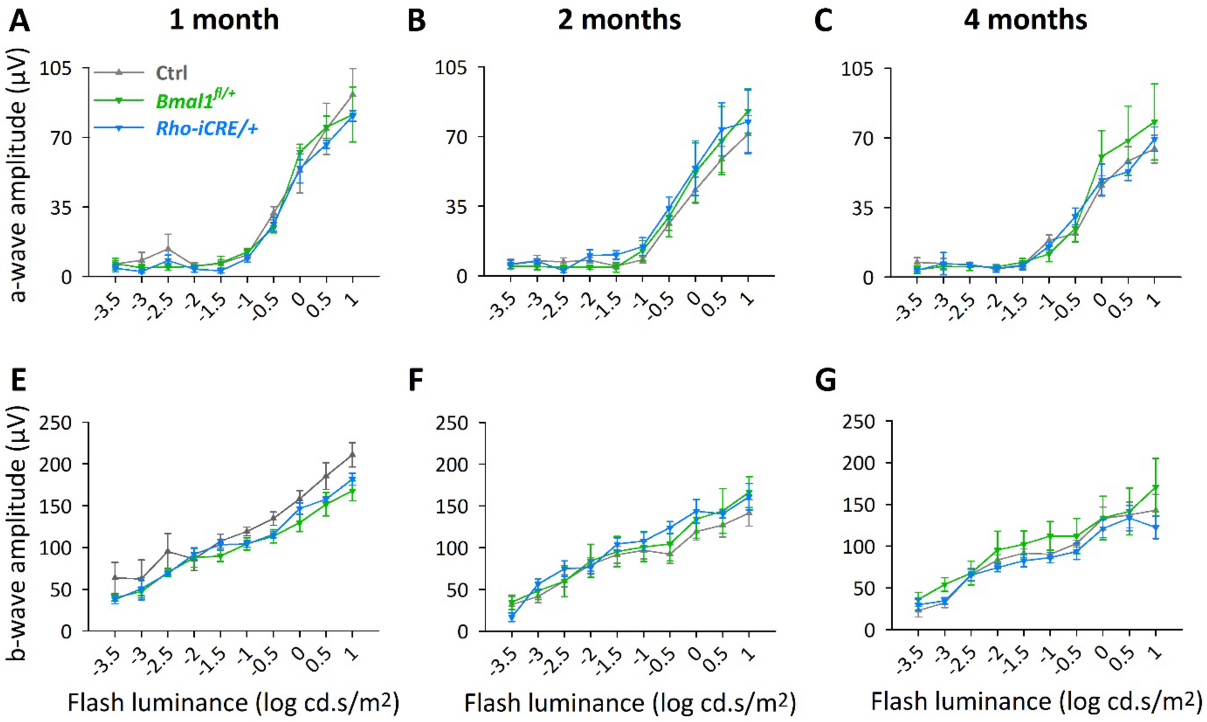

Supplementary Figure 1. Comparison of scotopic ERG amplitudes in Ctrl, *Bmal1*$^{fl/+}$ and *Rho-iCre/+* mice shows that the loss of one *Bmal1* allele does not impact light response between P40 and P180. Amplitudes of a- (A-D) and b- (E-H) waves are presented according to intensity of the light stimulus in Ctrl (*Bmal1*$^{fl/+}$ *; Rho-iCre/+*, gray) and *Bmal1*$^{fl/+}$ (green) and *Rho-iCre/+* (blue) mice. Comparison between genotypes was performed by Mixed effect model analysis (Suppl. Table 1). n = 4-7, n = 4-8 and n = 4-6/genotype at 1, 2 and 4 months, respectively.

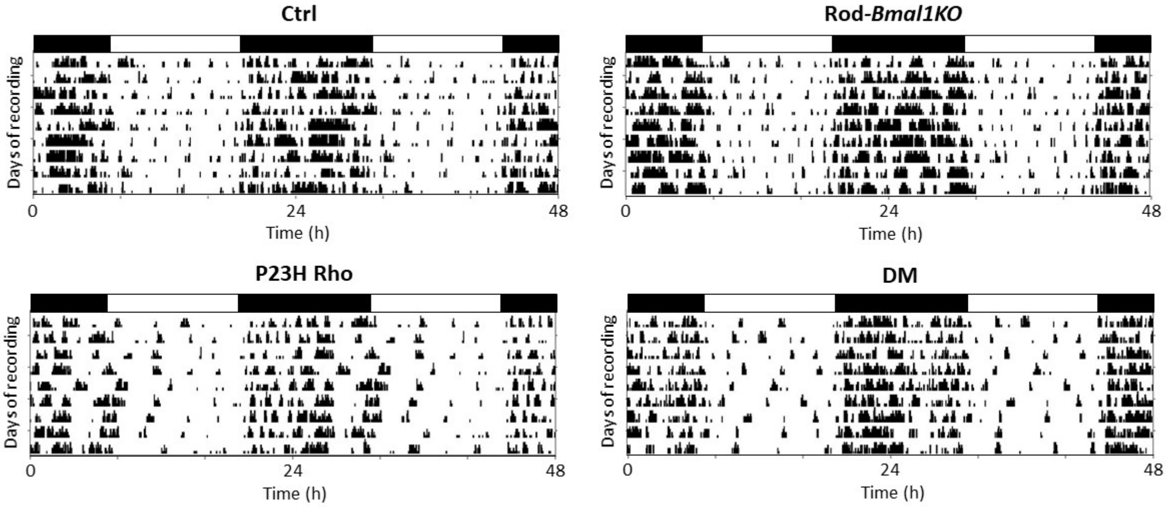

Supplementary Figure 2. Representative actograms of mice from the different genotype groups, exposed to a 12h/12h LD cycle, showing their capacity to entrain to this environmental synchronizer. Actograms were recorded as described : animals were housed in individual standard cages equipped with infrared detectors placed above the cage and linked to an automated recording system (CAMS, Circadian Activity Monitoring System, Lyon, France) (Salaberry et al., 2019). White and black bars indicate day and night periods respectively. No difference between genotypes (n = 4-6) was observed.

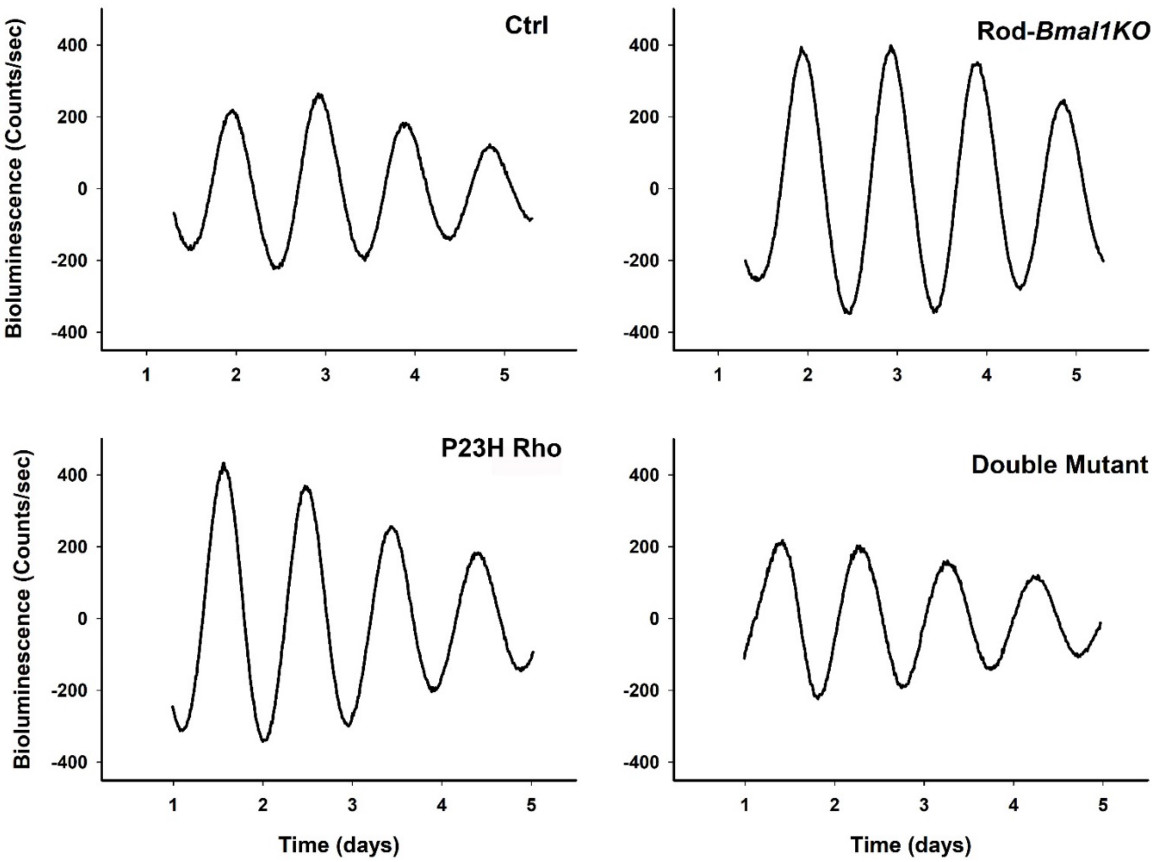

Supplementary Figure 3. Whole retina explants from the distinct genotype groups show intact PER2::Luciferase rhythmicity. PER2::Luciferase activity was recorded in real-time from retinal explants sampled from mice on the *Per2*$^{Luc/Luc}$ background, aged 1.5 to 3 months as previously described (Jaeger et al., 2015). Representative bioluminescence traces from Ctrl, rod-*Bmal1KO*, P23H Rho, and Double mutant retinas are shown.

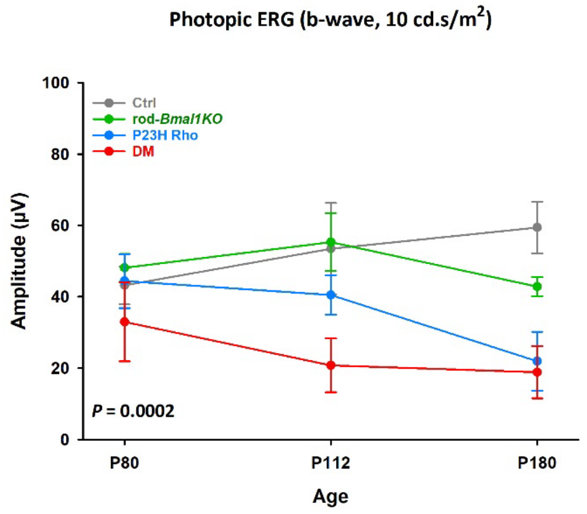

Supplementary Figure 4. Light-adapted (photopic) ERG was performed on all genotype groups at P80, P112 and P180. Amplitudes (means ± SEM) of the b-wave at 10 cd.s/m^2 are shown. There is a significant genotype effect (Mixed-effects model, *P* = 0.0002) (n=4-10 per genotype).

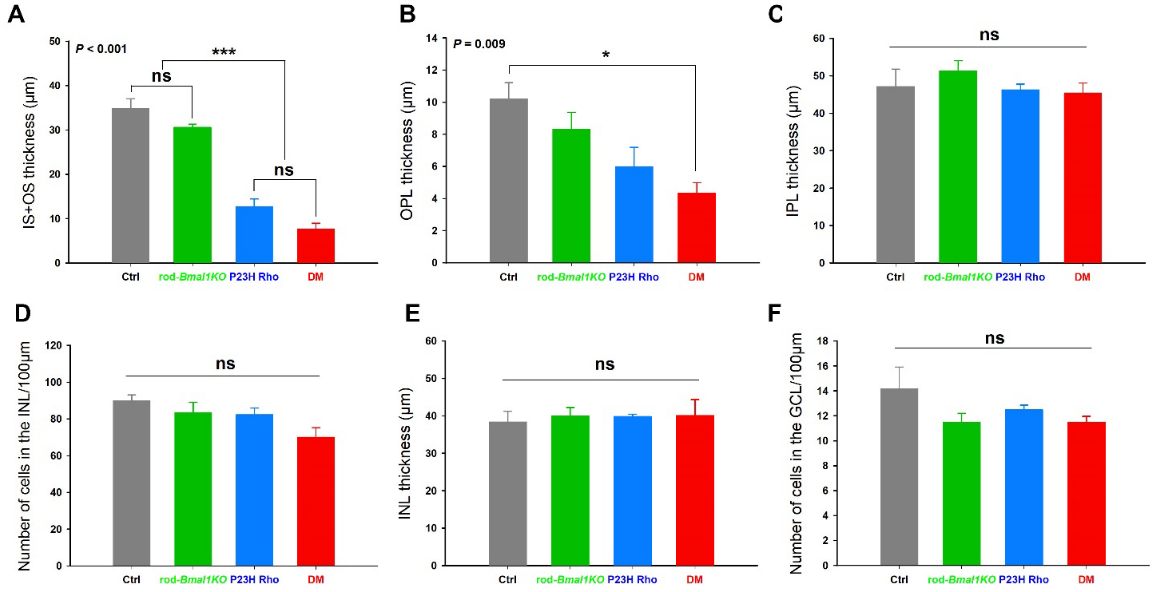

Supplementary Figure 5. Morphometric quantification of retinal sections at 750 µm from the optic nerve head in control (gray bar), rod-*Bmal1KO* (green bar), P23H Rho (blue bar), and DM (red bar). There is a significant genotype effect (*P*-value on the graph) for the thickness of the inner+outer segments (A) with significant difference between P23H Rho and DM with respect to Ctrl and rod-*Bmal1KO*, and outer plexiform layer (B) with DM being significantly distinct from Ctrl. No genotype effect was observed for inner plexiform layer thickness (C), INL density (D), INL thickness (E) and density in the ganglion cell layer (F). Data are presented as mean ± SEM; n = 3-4 mice/genotype. ns: non significant. *$P < 0.05$, ***$P < 0.001$.

A

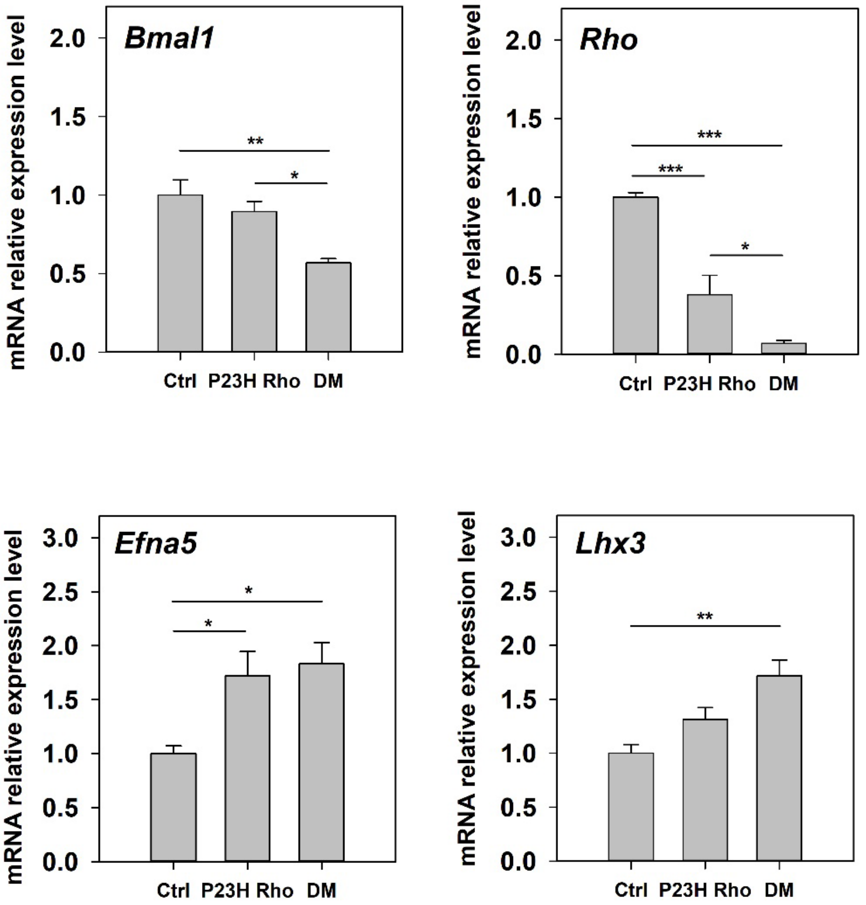

B

	Bmal1		*Rhodopsin*		*Efna5*		*Lhx3*	
	P23H Rho	DM	P23H Rho	DM	P23H Rho	DM	P23H Rho	DM
**RNASeq: Fold Change (vs Ctrl)**	0.68	0.61	0.25	0.07	1.17	1.66	1.15	1.71
**qPCR: Fold Change (vs Ctrl)**	0.89	0.56	0.38	0.07	1.72	1.83	1.31	1.71

Supplementary Figure 6. RT-qPCR analyses to validate the results from RNA-sequencing (same whole retina mRNAs) for the Ctrl, P23H Rho and DM retinas. (A) Relative expression was analysed for *Bmal1* to validate its decrease in the DM, for Rhodopsin, that confirmed major loss of rods, and *Efna5* and *Lhx3*, to confirm the processes of neurogenesis occurring in the DM. Expression patterns all showed significant genotype effect by One way ANOVA analysis ($P < 0.05$). Significant differences between genotypes, based on Holm-Sidak's post hoc analysis, are indicated: * P <0.05, ** P <0.01, *** P <0.001 (n = 4 per genotype). (B) Relative expression with respect to Ctrl samples as quantified following RNA sequencing and RT-qPCR, show the same tendency for all analysed genes.

Supplementary references

Jaeger C, Sandu C, Malan A, Mellac K, Hicks D, and Felder-Schmittbuhl MP (2015) Circadian organization of the rodent retina involves strongly coupled, layer-specific oscillators. Faseb J 29:1493-1504

Salaberry NL, Mateo M, and Mendoza J (2017) The clock gene Rev-Erbα regulates methamphetamine actions on circadian timekeeping in the mouse brain. Mol Neurobiol 54:5327-5334.
